# Kinetics of H_2_O_2_‑Induced Hydroxyl Radical Release by Carcinogenic Mineral Fibers Crocidolite, Chrysotile, and Erionite

**DOI:** 10.1021/acs.chemrestox.6c00003

**Published:** 2026-08-24

**Authors:** Elena Castellini, Alessandro F. Gualtieri

**Affiliations:** Department of Chemical and Geological Sciences, University of Modena and Reggio Emilia, Via Campi 103, Modena 41125, Italy

## Abstract

Hydroxyl radicals (^•^OH) display cyto- and genotoxicity and the ability to induce oxidative stress and chronic inflammation in vivo. In this work, we have monitored the kinetic profile of ^•^OH radical release from the carcinogenic mineral fibers crocidolite, chrysotile, and erionite at physiological pH (7.4). This was achieved via fluorescence spectroscopy, using coumarin-3-carboxylic acid as a semiquantitative probe. A kinetic model was developed to interpret the data. The formation of ^•^OH radicals is initiated by the binding of hydrogen peroxide (H_2_O_2_) to Fe^2+^ sites on the fibers, leading to oxidation to Fe^3+^ and release of ^•^OH through the Fenton reaction. ^•^OH release was also detected at acidic pH (4.1) under conditions that mimic the intracellular environment during fiber phagocytosis. Although the experiments were conducted in a simplified chemical system, some hypotheses on the activity of the investigated fibers in the extracellular alveolar environment can be done. The results suggest that ^•^OH generation is governed by a multistep mechanism that depends on the specific crystal-chemical nature of the fiber. At pH 7.4, crocidolite exhibits a radical production process which consists of a transition from a rapid oxidation of pristine surface Fe^2+^ to a continuous, long-term redox cycling of Fe^3+^. A similar long-term activity was observed for chrysotile, although masked at longer time scales by probe adsorption. In contrast, erionite, which lacks structural iron, releases ^•^OH at a slower rate but with a long-lasting process, likely driven by the reduction of Fe^3+^ present in nanoparticulate surface impurities. At long incubation times and also at acidic pH 4.1, the kinetic model and experimental evidence address the overlapping contribution of iron dissolution, indicating that homogeneous Fenton reactions complement the heterogeneous surface activity. These findings highlight distinct, time-dependent oxidative pathways for these mineral fibers, providing deeper insights into their specific biochemical mechanisms.

## Graphical abstract

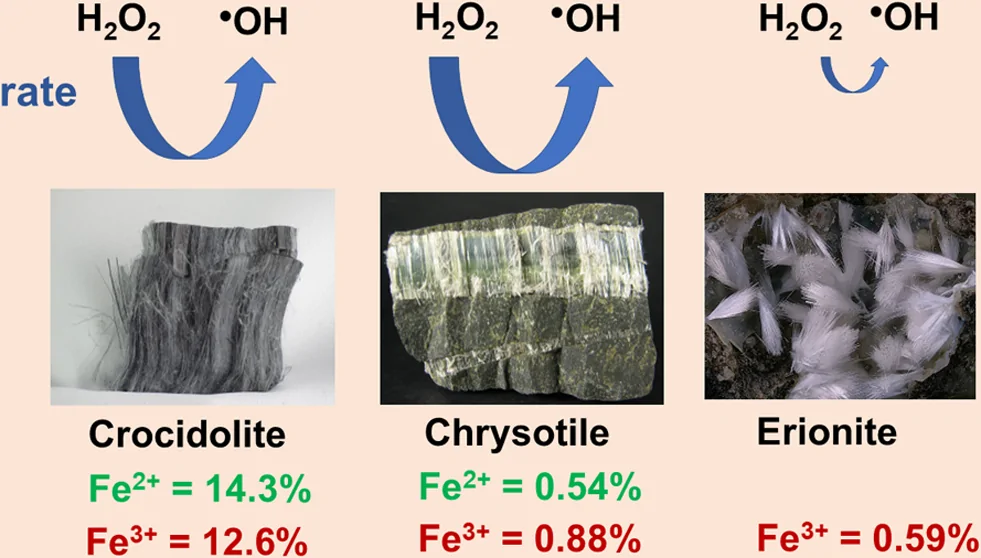

## Introduction

ROS (reactive oxygen species or reactive oxygen-containing species) is a collective term that includes the superoxide ion (O_2_^•–^), hydrogen peroxide (H_2_O_2_), hydroxyl radical (^•^OH), singlet oxygen (^1^O_2_), peroxyl radical (ROO^•^), alkoxyl radical (RO^•^), lipid hydroperoxide (LOOH), peroxynitrite (ONOO^–^), hypochlorous acid (HOCl), ozone (O_3_), and many other species.^1,2^ ROS cause inflammation but also regulate homeostasis in the human body and are involved in complex biochemical reaction pathways.^3^ They promote oxidative stress when their increase exceeds the antioxidant capacity of the cell.^3^ ROS have a paramount role in determining adverse effects of carcinogens *in vitro*/*in vivo*. They show at least three of the ten key characteristics (KC) assumed by the International Agency for Research on Cancer (IARC) to assess the carcinogenic potency of a chemical agent,^4^ in particular, (i) to be electrophilic or metabolically activated, (ii) to induce oxidative stress, and (iii) to induce chronic inflammation.

Carcinogenic mineral fibers like asbestos display ROS-related KCs. Free radical production is considered to be generally associated with carcinogenesis, considered here as a general term and not specifically addressed to mesotheliomagenicity or lung carcinogenicity only. In fact, ROS generation represents one of the fundamental mechanistic parameters identified by the IARC as contributing to the adverse effects prompted by carcinogens in vitro and in vivo. ROS production contributes to activating complex effects observed in vivo after the respiration of asbestos fibers that may create the microenvironment favoring carcinogenesis. The complex key mechanisms underlying malignant mesothelioma (MM) and lung cancer (LC) differ with major roles for chronic inflammation, oxidative stress, and DNA damage, promotion of a mutagenic microenvironment, tumor suppressor gene loss (e.g., CDKN2A, NF2, and BAP1), and release of nuclear protein HMGB-1 responsible for damage-associated molecular patterns (DAMPs) for MM and for chronic lung injury and inflammation, inflammatory cell recruitment, oxidative DNA damage, and genetic and epigenetic alterations for LC, respectively.^5–8^ ROS contribute to the pathogenesis of both diseases by acting as cyto- and genotoxic agents.^7,8^

In both in vitro and in vivo, ROS generation is usually called either “primary” if it is elicited at the surface of the fiber in contact with the cells or “secondary” if prompted by the inflammatory activity of the cell during frustrated phagocytosis of long, engulfed fibers in the deep respiratory apparatus. Primary ROS production at fiber surfaces is mediated by metals, namely, iron,^9^ and occurs via Fenton-catalyzed reactions: Fe^2+^ + H_2_O_2_ → Fe^3+^ + OH^–^ + ^•^OH.^10^ Production of ROS may also take place in the presence of Fe^3+^, which is known to be reduced by H_2_O_2_ to Fe^2+^, according to the reaction Fe^3+^ + H_2_O_2_ → Fe^2+^ + O_2_^•–^ + 2 H^+^.^11^ It should be noted that the generation of primary ^•^OH at the surface of the fibers interacting with a biomolecule requires the two to be in close contact, say within about 10 Å. This is because ^•^OH has a very short half-life in vivo (ca. 10^–9^ s) and a high reactivity. Hence, DNA damage from primary ^•^OH can only occur when the fiber is inside the nucleus of the cell. On the other hand, if iron can be mobilized from the fibers by chelators, such as citrate, the chelate complex can diffuse throughout the cell, catalyzing the formation of ^•^OH and thus damaging DNA.^10^

“Secondary” ROS are produced by alveolar or pleural macrophages during their attempts to clear long fibers, stimulating an “inflammatory burst” with the recruitment of other inflammatory cells such as neutrophils to the inflammation site.^6^ Excessive ROS production is responsible for the cellular oxidative stress. When oxidative stress is iron-dependent, cells may undergo ferroptosis, a type of programmed cell death genetically and biochemically distinct from apoptosis.^12^

“Primary” ROS, with special attention to ^•^OH, are measured in solids using several techniques. Electron paramagnetic resonance (EPR) spectroscopy is the most popular technique. In EPR, spin-trapping agents such as 5,5-dimethyl-1-pyrroline N-oxide (DMPO) are often used to form stable radical adducts to allow detection.^13^ Other methods involve fluorescence techniques, where hydroxyl radicals react with specific molecular probes to produce fluorescent products.^14^ Fourier-transform infrared (FTIR) spectroscopy can detect changes in the molecular structure of the solid, indicating hydroxyl radical presence.^15^ Chemiluminescence methods can also provide direct detection based on light emission from radical reactions.^16^ All these methods have advantages and disadvantages that may prevent their application to solid materials.

Indirect detection methods of the hydroxyl radical circumvent the problem of its short lifetime in vivo by linking the radical to a molecular probe able to produce a stable signal over a measurable period of time. For mineral fibers, in particular asbestos minerals, EPR spin-trapping analysis of hydroxyl radicals generated by the iron ions located on the fiber surface is the method of choice. In particular, the ^•^OH of the fibers in the presence of H_2_O_2_ can be quantified with DMPO as a spin-trapping agent using an EPR spectrometer and a split-ring resonator. A number of examples of application of this technique are available in the literature.^17–19^

In the last decades, fluorescence spectroscopy exploiting the coumarin probe (benzopyran-2-one, C) has been successfully applied to detect the signal produced by the 7-hydroxycoumarin compound (or umbelliferone),^20^ i.e., a product of the very fast reaction between the coumarin and the hydroxyl radical. The 7-hydroxycoumarin produces a fluorescence emission signal with a maximum at about 450 nm whose intensity is proportional to the hydroxyl radical amount. The fluorescence emission signal yield has been doubled using coumarin-3-carboxylic acid (3-CCA) because elimination of the hydroxylation in the C3 position increases the fluorescence of 7-hydroxylated coumarin-3-carboxylic acid (7-OH-3-CCA) 2-fold.^21,22^

In this work, the kinetics of the release of “primary” ^•^OH from carcinogenic mineral fibers relevant for industrial applications and health issues (crocidolite, chrysotile, and erionite) was systematically investigated for the first time, using fluorescence spectroscopy implemented with the coumarin-3-carboxylic acid spectrofluorimetric probe. The kinetic data were also analyzed in the light of the results of X-ray diffraction measurements performed on the solid fibers after the production of ^•^OH. This comparative kinetic analysis allowed stressing differences in the mechanisms of ^•^OH production by the different mineral species, which is central to clarifying the relationship between surface reactivity and fiber toxicity.

## Experimental Procedures

### Mineral Fibers and Chemical Reagents

The mineral fibers analyzed in this study are crocidolite (CRO), chrysotile (CHR), and erionite (ERI). Although all of them share common characteristics like being fibrous natural silicates and have been evaluated by the IARC as “carcinogenic for humans (group 1)”,^23^ their crystal-chemical assembly is very different.^24–26^ The structure description and the characterization of the three mineral fibers are reported in Supporting Information S1 together with the chemicals used.

### The 3-CCA–H_2_O_2_–Fluorescence Method

This method exploits the very quick reaction between the fluorescence probe coumarin-3-carboxylic acid (3-CCA) and ^•^OH to produce 7-hydroxycoumarin-3-carboxylic acid (7-OH-3-CCA), whose emission intensity at around 450 nm is directly related to the concentration of the released ^•^OH.^20^ A 10^–4^ M 3-CCA solution was prepared in a 10 mM phosphate buffer at pH 7.4 (Mettler Toledo pH meter, mod. SevenEasy) to simulate the physiological conditions and achieve a careful pH control, which is necessary due to the high pH sensitivity of the fluorescence spectrum of the 7-OH-3-CCA. The fluorescence spectrum of 10^–4^ M 7-OH-3-CCA in 10 mM phosphate buffer at pH 7.4 and in 10 mM acetate buffer at pH 4.1 was measured in order to know the effect of pH on the spectrum (Supporting Information S2 and Figure S1).^21^ The low 3-CCA concentration was chosen to avoid the problem of the quenching of the emission signal of the produced 7-OH-3-CCA.^20^ A 10^–2^ M H_2_O_2_ solution was prepared by diluting 21.8 μL of the pure reagent in a 25 mL flask. Batches of samples were prepared weighing 5 mg for each fiber (CRO, CHR, and ERI) in a 1.5 mL Eppendorf test tube. All kinetic measurements were performed under aerated conditions. This design ensured continuous gas exchange and complete oxygen saturation throughout the entire incubation period, thereby preventing any artifacts related to oxygen depletion or variable oxygen concentration.

A 1 mL portion of 3-CCA solution was added to each Eppendorf containing the fiber, followed by 20 μL of the 10^–2^ M H_2_O_2_ solution to reach the final H_2_O_2_ concentration of 2 × 10^–4^ M. The injection of H_2_O_2_ was considered the starting point of the reaction of the formation of the hydroxyl radical by the fibers and of the 7-OH-3-CCA product as well, since the reaction between the hydroxyl radical and the spectrofluorimetric probe 3-CCA is extremely fast. The above suspensions were mixed, shaken by hand for 30 s, and placed in a thermostat (FALC, mod FA 90) at 37 °C for different time frames: 1, 2, 3, 6, 14, 14, 24, 38, 48, 72, 120, 192, 264, 528, 720, and 792 h (h). At each selected time interval, the suspensions were removed from the thermostat and centrifuged at 12,800 rpm for 20 min (centrifuge Labnet, mod 24D); the supernatant containing the reaction products was separated with a pipet from the fibers and eventually analyzed by fluorescence (Jasco instrument, FP-6200 model) and UV–vis techniques (Jasco instrument, V-570 model). The solid fibers were washed with distilled water and taken to the X-ray powder diffraction (XRPD) analysis (PANalytical ’XPert Pro Diffractometer). The same procedure has been applied to the three fibers also in the presence of 10^–3^ M 2,2,6,6-(tetramethylpiperidin-1-yl)­oxyl (TEMPO), a powerful radical scavenger used to provide chemical evidence that the hydroxylation of 3-CCA in our system is driven by free hydroxyl radicals.^27,28^ The measurements of the intensity of the fluorescence emission at 446 nm (*I*_em 446 nm_) of 7-OH-3-CCA produced by the fibers as a function of time were repeated three times for each fiber, and the reported data are the corresponding mean values.

### Calibration Curves of 7-OH-3-CCA

To quantify the production of the hydroxyl radicals by the fibers, the calibration curves were performed at pH 7.4 (Supporting Information S3 and Figure S2a) and 4.1 (Supporting Information S3 and Figure S2b), reporting the fluorescence emission intensity of the signal at 446 nm in the spectrum of 7-OH-3-CCA as a function of its concentrations. 7-OH-3-CCA was employed to prepare the “mother” solutions.

### H_2_O_2_-Refill Test

A 5 mg amount of CRO, CHR, and ERI fibers was weighed in a 1.5 mL Eppendorf test tube. A 1 mL portion of phosphate buffer solution at pH 7.4 was then added together with H_2_O_2_ at a final concentration of 2 × 10^–4^ M (as for the 3-CCA–H_2_O_2_–fluorescence method, but without the 3-CCA reagent). The three suspensions were mixed and shaken for 30s and then placed for 72 h in the thermostat (FALC, mod FA 90) at 37 °C. After 72 h, the H_2_O_2_-treated solids were separated by centrifugation at 12,800 rpm for 20 min (centrifuge Labnet, mod 24D); the liquids were discharged, and the 3-CCA–H_2_O_2_–fluorescence method was applied to the solids. In particular, 1 mL of 10^–4^ M 3-CCA was added in each Eppendorf containing the H_2_O_2_-treated fiber together with H_2_O_2_ at a 2 × 10^–4^ M final concentration. After 72 h, the suspensions were centrifuged, and the solutions were taken to the fluorescence measurements to evaluate the presence and detect the concentration of the 7-OH-3-CCA species.

### Tests with the 3-CCA–H_2_O_2_–Fluorescence Method at Different pHs

To simulate the fate of a fiber in contact with the acidic environment of an intracellular phagolysosome produced during alveolar phagocytosis of the macrophages, a set of measurements at different pH values was executed. The 3-CCA–H_2_O_2_–fluorescence method was applied to the three fibers first at pH 7.4 for 48 h, then at pH 4.1 for 24 h, and then again at pH 7.4 for 5 h. A 10^–4^ M 3-CAA solution was prepared in a 10 mM acetate buffer at pH 4.1 (Mettler Toledo pH meter, mod SevenEasy). All the other experimental conditions remained the same as at pH 7.4. After the first set of experiments at pH 7.4, the separated solids were washed once with distilled water by centrifugation at 12,800 rpm for 20 min before being added to 3-CCA at pH 4.1 and H_2_O_2_. Instead, the same fibers were washed two times with distilled water before being added to 3-CCA at pH 7.4 and H_2_O_2_ for the second time, as the buffer at pH 4.1 is more persistent to be removed. Two series of samples were prepared, and the results were reported as the mean values.

### Contribution of Released Iron to 7-OH-3-CCA Production

Batches of samples were prepared to monitor iron release into the 3-CCA solution during fiber incubation and to evaluate its capacity to generate 7-OH-3-CCA. This provided an estimation of the contribution of the homogeneous Fenton reaction to the overall hydroxyl radical production. For each sample, 10 mg of fibers was placed into a 15 mL Falcon tube, followed by the addition of 2 mL of a 10^–4^ M 3-CCA solution prepared in a 10 mM phosphate buffer (pH 7.4). The suspensions were mixed, shaken manually for 30 s, and then incubated in a thermostat (FALC, mod FA 90) at 37 °C. After 1, 3, and 14 days (d), the suspensions were removed from the thermostat; the supernatant was separated from the fibers using a pipet, and the resulting solutions were centrifuged at 12,800 rpm for 20 min (centrifuge Labnet, mod 24D); the solutions obtained after centrifugation were divided into two aliquots. The first aliquot (1 mL) was transferred to an Eppendorf tube, where H_2_O_2_ was added according to the 3-CCA–H_2_O_2_–fluorescence method; this was done to detect the production of 7-OH-3-CCA, which indicates the presence of ^•^OH via a homogeneous Fenton reaction. The second aliquot, with a volume of less than 1 mL, was prepared for I.C.P.-O.E.S. (Inductively Coupled Plasma—Optical Emission Spectroscopy) analysis to determine the concentration of released iron. The exact volume of this second aliquot was determined by weight; it was then diluted to 5 mL, adding also 5% v/v of HNO_3_.

The same procedure (3-CCA–H_2_O_2_–fluorescence method and ICP-OES measurements) was repeated for the fibers incubated with 3-CCA solution for 1 d at pH 4.1.

A number of normalization methods (mass normalization, fiber number normalization, fiber-specific surface normalization, and many more) are used in toxicological studies. Mass-based normalization was adopted to evaluate the intrinsic chemical reactivity of the mineral fibers on an equal weight basis. This approach ensures a consistent concentration of the mineral matrix and its constituent ROS-inducing transition metals across all of the tested samples. Furthermore, for cell-free radical generation assays, mass dosing provides a highly reproducible benchmark, avoiding the experimental uncertainties and artifacts linked to fiber counting or surface area variations in highly porous mineral phases.^29^

### Instrumental Techniques

The pH of the solutions separated from the fibers was checked with a pH meter (Mettler Toledo pH meter, mod SevenEasy) to make sure that the physiological pH value of 7.4 remained constant. UV–vis and fluorescence measurements were performed to detect 3-CCA and 7-OH-3-CCA on the solutions (Supporting Information S4 and Figure S3), while X-ray powder diffraction (XRPD) was used to characterize the resulting solids after contact with the solutions.^30^ The I.C.P.-O.E.S. technique was used to measure the iron release onto leachate solution from the three fibers at different times (all instrumental details are reported in Supporting Information S4).

## Results and Discussion

### Fluorescence Measurements

The fluorescence spectra were acquired on the solutions separated from the three fibers reacted with 3-CCA and H_2_O_2_ for different times at pH 7.4 (Supporting Information S5 and Figure S4a–c). The shape of the spectra strictly resembles that of 7-OH-3-CCA at pH 7.4, showing a prominent peak with a maximum at 446 nm (Supporting Information S2 and Figure S1), thus uniquely identifying the reaction product with the 7-OH-3-CAA compound. This finding indicates that ^•^OH radicals are formed under the experimental conditions used here and react with 3-CCA to form 7-OH-3-CCA.

To unambiguously confirm that the formation of 7-OH-3-CAA is mediated by free hydroxyl radicals ^•^OH, competitive quenching experiments were performed, introducing into the systems above-described 2,2,6,6-(tetramethylpiperidin-1-yl)­oxyl (TEMPO) as a powerful free-radical scavenger. Upon the addition of TEMPO, a near-complete suppression of the 7-OH-3-CAA fluorescence signal was observed (Supporting Information S6 and Figures S5a–c). Because TEMPO reacts with transient free radicals at diffusion-controlled rates (*k* ≈ 10^9^ M^–1^ s^–1^) via radical coupling, this dramatic inhibition provides definitive chemical evidence that the hydroxylation of 3-CCA in our system is strictly driven by free hydroxyl radical pathways.

The fluorescence emission intensity of 7-OH-3-CCA at 446 nm is directly related to the amount of generated ^•^OH. Thanks to the fast reaction kinetics between the 3-CCA probe and the hydroxyl radicals, this signal enables the real-time detection of ^•^OH production.

Figure 1 shows the comparison of the time course of the intensity of the fluorescence emission signal at 446 nm (*I*_em 446 nm_) of the 7-OH-3-CCA species produced by the CRO, CHR, and ERI fibers.

**Figure 1:**
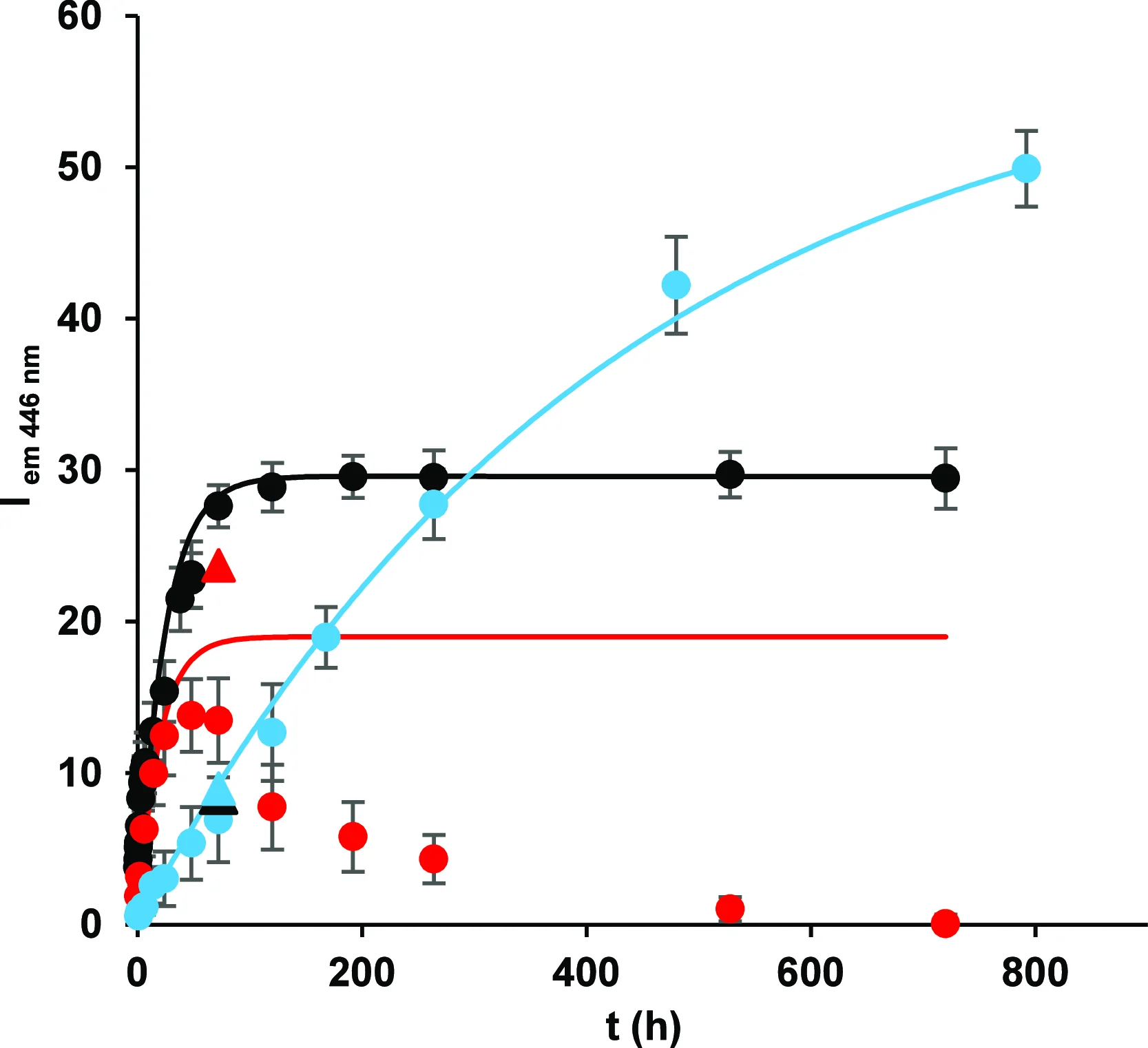
Time course of the intensity of the fluorescence emission signal at 446 nm (*I*_em 446 nm_) of the 7-OH-3-CCA species produced by CRO (•), CHR (red •), and ERI (blue •) fibers reacted with 3-CAA (10^–4^ M) and H_2_O_2_ (2 × 10^–4^ M) at pH 7.4. Solid lines (CRO —; CHR red —; ERI blue —) are the corresponding best-fitting curves to eq 24 (vide infra) obtained with SigmaPlot version 12.3. For CHR only, the data until 24 h have been included in the best fit (see below). At *t* = 72 h, the refill data have also been inserted (CRO ▲; CHR red ▲; ERI blue ▲).

All the fibers show an emission intensity that increases with time, but with significant differences. In particular, the CRO signal steeply grows and, after 72 h, reaches a plateau, while the ERI signal increases much more slowly without leveling off. CHR shows a complex behavior which resembles that of CRO until about 48 h, but the signal intensity decreases thereafter. The results of the UV–vis measurements performed on the same samples immediately after the fluorescence measurements suggest an explanation for the behavior of CHR. In fact, while CRO and ERI do not show any adsorption process (Supporting Information S7 and Figure S6a,b), CHR does, and adsorption increases with time.

Figure 2 shows the time course of the UV–vis spectra of the 7-OH-3-CCA produced by the ^•^OH overlapping with that of the 3-CCA, along with that of the starting 3-CCA solution. The intensity of the absorption band with a maximum at 291 nm (typical of the aromatic ring) related to the concentration of the 3-CCA decreases with time with respect to the starting 3-CCA solution (Figure 2), indicating an ongoing adsorption process probably involving both 3-CCA and 7-OH-3-CCA on the CHR fiber. The adsorption ability of CHR can be explained by its larger specific surface area with respect to CRO and ERI and the fact that, in distilled water, the zeta potential of CHR is positive while those for CRO and ERI are negative^26^ and the carboxylic groups of 3-CCA and 7-OH-3CCA are deprotonated at pH 7.4. Thus, the kinetic analysis for the CHR data has been performed only up to 24 h. Afterward, estimation of the ^•^OH produced is not reliable because adsorption alters the concentration of 7-OH-3-CCA and the nature of the CHR surface. Such adsorption, although to a lower extent, may also be responsible for underestimation of the radicals produced for shorter reaction times. Notably, the continuous increase in the net fluorescence signal up to 48 h (Figure 1), despite the adsorption process being already active at earlier time points (e.g., 6 h, Figure 2), reflects the dynamic competition between active radical generation and surface sequestration. During the initial stages of the reaction, the robust catalytic generation of ^•^OH by the highly active surface Fe^2+^ centers outpaces the rate of physical removal of the probe from the bulk solution, resulting in a net positive accumulation of the fluorescent adduct. It is only after extended incubation times, when a critical threshold of surface coverage is reached, that this progressive passivation (“blanketing” effect) dominates, ultimately driving the anomalous drop in the solution fluorescence. The reliability of the fit of the CHR kinetic data (red line in Figure 1) is nicely confirmed by the very good match with the kinetic curve reported in Figure 4a of Walter et al.^19^ for the decomposition of H_2_O_2_ in contact with pristine (raw) CHR fibers.

**Figure 2:**
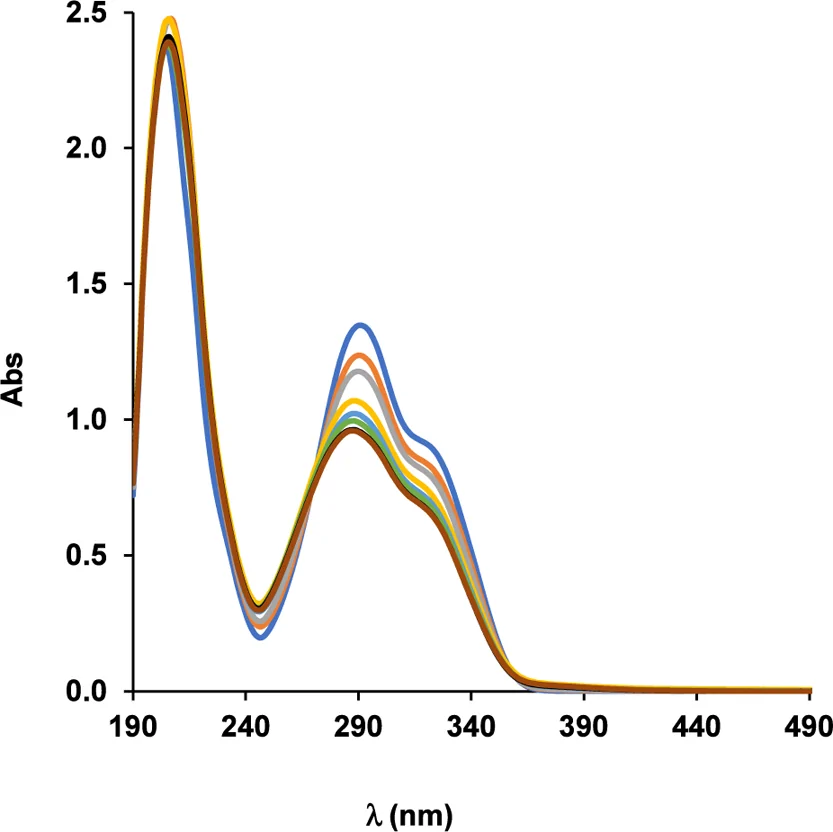
UV–vis spectrum of the starting 3-CCA 10^–4^ M solution (blue) and of the solutions separated from the suspensions made by CHR plus 10^–4^ M 3-CCA and 2 × 10^–4^ M H_2_O_2_ at 1 (orange), 2 (gray), 6 (yellow), 14.5 (light blue), 24 (green), 48 (black), and 72 (brown) h.

### Kinetic Model

To model the accumulation of radical species over time and provide a mathematical framework for comparing the three minerals, a kinetic expression was derived based on a formal heterogeneous Langmuir–Hinshelwood catalytic scheme. Production of ^•^OH by the mineral fibers is assumed to proceed via two sequential steps: (I) the reversible adsorption/desorption of H_2_O_2_ onto surface iron centers involving the displacement of pristine surface hydroxyl metal ligands and (II) the surface chemical reaction leading to the catalytic decomposition of the coordinated peroxide and the release of hydroxyl radicals with the restoration of the metal hydroxide bonds.^24^

I $$surface-{Fe}^{2+}-OH+H_{2}O_{2}\overset{k_{1}}{\underset{k_{-1}}{⇄}}surface-{Fe}^{2+}-H_{2}O_{2}+{OH}^{-}$$

surface−Fe2+−H2O2→k′catsurface−Fe3+−OH+OH• II

Here, *k*_1_ is reserved for the forward adsorption rate constant of H_2_O_2_ onto the surface, *k*_–1_ represents the backward desorption rate constant, and *k*′_cat_ denotes the intrinsic surface reaction rate constant. The first step is a fast pseudoequilibrium in which the kinetic constant of the backward reaction (*k*_–1_) is much higher than that of the direct reaction (*k*_–1_ ≫ *k*_1_). This fact reflects the higher affinity of Fe^2+^ centers toward the pristine OH^–^ ion with respect to the reagent H_2_O_2_. The second step is a quantitative reaction whose kinetic constant (*k*′_cat_) is smaller than *k*_–1_ (*k*_–1_ ≫ *k*′_cat_). The reaction I goes backward faster than it proceeds in step II. By assuming a fast pseudoequilibrium for step (I), the adsorption–desorption steps are considered to reach equilibrium much faster than the chemical transformation proceeds.

The active-site-normalized adsorption rate (v_adsorption_) of H_2_O_2_ on the surface of the fiber (step I) is proportional to the kinetic constant of the direct reaction, to the concentration of the H_2_O_2_ reagent, and to the fraction of the free iron sites on the fiber surface. It can be expressed as

1 $$v_{adsorption}=k_{1}\left[H_{2}O_{2}\right]\left(1-\theta \right)$$

where θ is the fractional surface coverage, defined rigorously as the fraction of active surface iron sites occupied by H_2_O_2_.

The corresponding desorption rate (v_desorption_) of H_2_O_2_ from the surface is given by two contributions: (i) the backward reaction in step I, which restores the starting surface and the H_2_O_2_ reagent and is proportional to the kinetic constant *k*_–1_ and to the fraction of iron sites bound to H_2_O_2_ (occupied active iron sites), and (ii) the reaction which leads to the ^•^OH production, which is proportional to the kinetic constant *k*′_cat_ and to the fraction of occupied iron sites:

2 $$v_{desorption}=k_{-1}\theta +k_{cat}^{\prime }\theta$$

Being *k*′_cat_ ≪ *k*_–1_ for the hypothesis of the fast equilibrium, the term *k*′_cat_θ can be neglected:

3 $$v_{desorption}=k_{-1}\theta$$

The hypothesis of the fast equilibrium in step I implies that

4 $$v_{adsorption}=v_{desorption}$$

5 $$k_{1}\left[H_{2}O_{2}\right]\left(1-\theta \right)=k_{-1}\theta$$

from (5), it is possible to derive the value of θ:

6 $$\theta =\frac{k_{1}\left[H_{2}O_{2}\right]}{k_{1}\left[H_{2}O_{2}\right]+k_{-1}}$$

Under this framework, the fractional surface coverage (θ) is therefore governed by the Langmuir isotherm.

The net rate of ^•^OH radical production (v) is directly proportional to the amount of H_2_O_2_ adsorbed on the active sites and can be written in a lumped form commonly used in heterogeneous kinetics as

v=d[OH•]dt=kcatθ 7

Crucially, eq 7 does not equate fractional coverage directly with the absolute radical production rate. Rather, the total density of reactive surface iron sites ([S]_0_) is treated as a constant under our experimental conditions and is embedded within the parameters governing the forward surface processes. Specifically, the apparent surface reaction constant (*k*_cat_) scales with the total site density as follows:

8 $$k_{cat}=k_{cat}^{\prime }{\left[S\right]}_{0}$$

where *k*′_cat_ represents the intrinsic, site-independent kinetic constants.

The rate of disappearance of the peroxide reagent from the bulk solution is dictated by its surface reaction and can be formally expressed as

$$-\frac{d\left[H_{2}O_{2}\right]}{dt}=k_{cat}^{\prime }{\left[S\right]}_{0}\theta =k_{cat}\frac{k_{1}\left[H_{2}O_{2}\right]}{k_{1}\left[H_{2}O_{2}\right]+k_{-1}}$$ 9

Equation 9 clearly deviates from first-order behavior as the concentration of H_2_O_2_ increases and the surface approaches saturation (θ → 1). However, the system can be treated under a low surface coverage limit (θ ≪ 1) when [H_2_O_2_] is sufficiently small (our case) and *k*_–1_≫ *k*_1_. Physically, this condition is met due to the significantly higher thermodynamic affinity of the surface Fe^2+^ centers for the native OH^–^ ions compared to the H_2_O_2_ reagent. Under this linear regime, the term *k*_1_[H_2_O_2_] in the denominator becomes negligible compared to *k*_–1_, and the rate expression simplifies to

10 $$-\frac{d\left[H_{2}O_{2}\right]}{dt}=\frac{k_{1}k_{cat}}{k_{-1}}\left[H_{2}O_{2}\right]=k_{obs}\left[H_{2}O_{2}\right]$$

where *k*_obs_ is the apparent first-order decay constant of H_2_O_2_ in solution.

In our study, the experimental concentrations of [H_2_O_2_] were strictly maintained within this linear, low-coverage regime (2 × 10^–4^ M). Consequently, the depletion of the reagent follows pseudo-first-order kinetics over time, described by the exponential decay law:

11 $$\left[H_{2}O_{2}\right]={\left[H_{2}O_{2}\right]}_{0}e^{-k_{obs}t}$$

Rather than an arbitrary empirical fitting parameter, *k*_obs_ is therefore a complex operational constant, incorporating the intrinsic surface reactivity, the thermodynamic adsorption affinity, and the total active site density [S]_0_:

$$k_{obs}=\frac{k_{1}k_{cat}}{k_{-1}}=\frac{k_{1}{k^{\prime }}_{cat}}{k_{-1}}{\left[S\right]}_{0}$$ 12

As in step (I), *k*_–1_ ≫ *k*_1_, and, moreover, in our experimental case, [H_2_O_2_] is very low, so the term *k*_1_[H_2_O_2_] can be neglected in the denominator of eq 6:

13 $$\theta =\frac{k_{1}\left[H_{2}O_{2}\right]}{k_{-1}}$$

and therefore eq 13 can we written as

14 $$\theta =\frac{k_{1}{\left[H_{2}O_{2}\right]}_{0}e^{-k_{obs}t}}{k_{-1}}$$

The constant *A*_0_ can be fixed as

15 $$A_{0}=\frac{k_{1}{\left[H_{2}O_{2}\right]}_{0}}{k_{-1}}$$

Equation 14 becomes

16 $$\theta =A_{0}e^{-k_{obs}t}$$

The rate of the production of the ^•^OH radicals is represented by

v=d[OH•]dt=kcatθ=kcatA0e−kobst 17

The constant B_0_ can be fixed as

18 $$B_{0}=k_{cat}A_{0}$$

Equation 17 can be written as

d[OH•]dt=B0e−kobst 19

Integrating both members in eq 19 upon the separation of the variables yields

∫d[OH•]=B0∫e−kobstdt 20

[OH•]=B0e−kobst(1−kobs)+constant 21

It is possible to derive the value of the constant considering eq 21 for *t* = 0 and thus [^•^OH] = 0:

22 $$constant=B_{0}\left(\frac{1}{k_{obs}}\right)$$

Equation 21 becomes

[OH•]=B0e−kobst(1−kobs)+B0(1kobs)=B0(1kobs)(1−e−kobst) 23

for *t* → ∞, [^•^OH] becomes [^•^OH]_lim_, thus for *t* → ∞, eq 23 provides the value of [^•^OH]_lim_:

[OH•]lim=B0(1kobs) 24

[OH•]=[OH•]lim(1−e−kobst) 25

Equation 25 can be rearranged as

[OH•][OH•]lim=(1−e−kobst) 26

Crucially, the 3-CCA–H_2_O_2_–fluorescence method serves as an operational, semiquantitative proxy tool rather than an absolute stoichiometric counter. This approach acts as a semiquantitative comparative proxy tool where the observed fluorescence intensity (*I*_em_) is proportional to the accumulated concentration of the tracking adduct (7-OH-3-CCA) in solution. Under heterogeneous Fenton-like conditions, a strict 1:1 proportionality between the total amount of ^•^OH radicals generated and the resulting fluorescent 7-OH-3-CCA adduct may not hold due to the unavoidable formation of other nonfluorescent hydroxylation products, possible complexation phenomena, or multistep oxidation pathways.^20^

Nevertheless, because all investigated mineral fibers were tested under strictly identical experimental conditions, including initial probe concentration, pH, temperature, and buffer composition, any systematic deviation or nonlinearity affects the samples equally. Consequently, the kinetic branching ratio between these competing pathways remains constant throughout the reaction course for all fibers, ensuring a reliable comparative analysis. Therefore, the relationship can be rigorously expressed via a constant yield Φ, where [7-OH-3-CCA] = Φ [^•^OH]. Since Φ remains invariant under these controlled conditions, this scaling factor behaves as a constant multiplier in the integrated rate law. Consequently, when fitting the exponential approach to the plateau, the scaling factor affecting the pre-exponential factor does not influence the exponential decay constant *k*_obs_. Therefore, the semiquantitative nature of the absolute concentration does not affect the mathematical determination of the reaction order, the kinetic profiles, or the relative comparison of the rate constants among the three fibers. Within this standardized setup, the accumulation of the fluorescent signal directly reflects the relative kinetic fingerprint of each fiber, allowing the concentration ratio [OH•][OH•]lim to be confidently mapped onto the operational signal ratio $\frac{I_{em}}{I_{em\lim}}$ for internal comparative purposes.

27 $$\frac{I_{em}}{I_{em\lim}}=\left(1-e^{-k_{obs}t}\right)$$

28 $$I_{em}=I_{em\lim}\left(1-e^{-k_{obs}t}\right)$$

It is fundamental to underscore that *I*_em lim_ and *k*_obs_ are operational, lumped kinetic parameters. They do not resolve single microscopic steps at the single-site level but rather capture the net macroscopic result of radical generation, surface redox cycling (Fe^2+^/Fe^3+^), and progressive surface evolution (such as probe adsorption or secondary precipitation) under the specific experimental conditions. The experimental data were analyzed with this lumped operational model (eq 28) via best-fitting regressions using SigmaPlot version 12.3. The resulting effective parameters (*I*_em lim_ and *k*_obs_) are compiled in Table 1. For CHR, the regression was strictly restricted to data points obtained below 25 h to mitigate the mathematical artifacts introduced by the severe probe adsorption and iron reprecipitation phenomena discussed previously. The reliability and goodness of fit of the kinetic model were evaluated based on the correlation coefficients (*R* ^2^) and the statistical significance (*p*-values) associated with the estimated parameters, namely, the limiting emission intensity (*I*_em lim_) and the observed rate constant (*k*_obs_). As summarized in Table 1, all fitted parameters exhibit high statistical significance, with *p*-values consistently well below the standard threshold of 0.05 (*p* < 0.0001 for nearly all parameters and *p* = 0.0007 for the *k*_obs_ of CHR). This confirms that the extracted kinetic parameters are highly reliable and not artifacts of experimental noise. Furthermore, the model demonstrates a strong capacity to describe the experimental data across all tested samples. The *R* ^2^ values range from 0.9071 for CRO to an excellent 0.9950 for ERI. The remarkably high *R* ^2^ observed for ERI, combined with the narrow standard errors for both *I*_em lim_ (59.0 ± 1.8) and *k*_obs_ (0.0024 ± 0.0001 h^–1^), underscores the robustness of the fit for this system.

**Table 1:** Values of the *I*_em lim_ and *k*_obs_ Parameters Obtained Applying the Best Fit of eq 24 with SigmaPlot Version 12.3 to the Data Reported in Figure 1

	*I*_em lim_	*p* (*I*_em lim_)	*k*_obs_ (h^–1^)	*p* (*k*_obs_)	*R* ^2^
CRO	29.6 ± 1.9	<0.0001	0.0429 ± 0.0054	<0.0001	0.9071
CHR	19.0 ± 2.2	<0.0001	0.0522 ± 0.0055	0.0007	0.9351
ERI	59.0 ± 1.8	<0.0001	0.0024 ± 0.0001	<0.0001	0.9950

The apparent kinetic constants (*k*_obs_) of CRO and CHR are remarkably similar and approximately 20-fold higher than that calculated for ERI, although for CHR, the model describes exclusively the first 25 h before surface passivation takes over. The limiting value of the proxy emission intensity (*I*_em lim_), along with the distinctly different shapes of the kinetic profiles, highlights significant variations in the maximum comparative radical-generating capacity and the sustained duration of long-term catalytic activity among the three mineral fibers.

### H_2_O_2_-Refill Test

These measurements were aimed at assessing if the production of ^•^OH observed for longer times (Figure 1) must be ascribed to the consumption of the H_2_O_2_ reagent or to the inactivation of the Fe^2+^ centers in the fibers. For this reason, the fibers were treated for 1 week only with H_2_O_2_ without 3-CCA: in this way, the radical-producing reagent H_2_O_2_ is present, while the 3-CCA, which may adsorb on CHR, is absent. After this treatment, the H_2_O_2_-treated fibers were tested with the 3-CCA–H_2_O_2_–fluorescence method at t = 72 h to check if they are still able to produce ^•^OH or are inactivated, i.e., if the radical production can restart or not. The points corresponding to the refill test have been inserted in Figure 1 (with the symbols CRO ▲, CHR red ▲, and ERI blue ▲).

The results show that the treatment with H_2_O_2_ for 168 h at the concentration employed in the 3-CCA–H_2_O_2_–fluorescence method (2 × 10^–4^ M) leaves the capacity of ERI to produce radicals unchanged, while it decreases that of CRO and increases that of CHR (Figure 1).

These results provide an explanation for the behavior of the three fibers in Figure 1. The capacity of CRO to produce radicals ends after a week just because all the H_2_O_2_, which in these conditions acts as a limiting reagent, has been consumed. Consistently, after a week of treatment with H_2_O_2_, introduction of additional H_2_O_2_ into the system is able to make the production of ^•^OH to start again, although to a lesser extent with respect to the starting condition. For CRO, exposure to H_2_O_2_ does not induce or modify any adsorption capacity; the UV–vis spectra of 3-CCA solutions in contact with H_2_O_2_-treated CROC remained perfectly stable over time, showing no decrease in absorbance. The behavior of CHR is surprising because the production of hydroxyl radicals increases after the H_2_O_2_ treatment. This result confirms that the progressive decrease in the radical production observed in the standard kinetic curve (Figure 1) is heavily dictated by probe-surface interactions and the complex dynamic of incongruent iron dissolution followed by secondary phase reprecipitation, which acts by progressively masking the active surface sites or trapping the fluorescent adduct.

During the 168 h H_2_O_2_ treatment in the absence of the 3-CCA probe, CHR undergoes surface etching and structural evolution without the immediate interference or complexation of the organic molecule on the mineral interface. This pristine surface evolution likely enhances the bioavailability of reactive iron sites, explaining why CHR actually increases the comparative production of ^•^OH 2-fold when the probe is finally introduced.

The behavior of ERI after the H_2_O_2_ treatment is as expected. Figure 1 shows that it keeps producing radicals after 792 h. Thus, it is reasonable that after the H_2_O_2_ treatment, ERI still possesses many Fe^2+^ sites capable of producing hydroxyl radicals, and therefore, after 72 h of contact with 3-CAA and H_2_O_2_, it maintains the usual mechanism of radical production.

Iron Release in 3-CCA Solutions and the Role of the Homogeneous Fenton Reaction in ^•^OH Radical Production

The results concerning the iron release from the three mineral fibers, determined by the I.C.P.-O.E.S. technique (Table 2), demonstrate that the leaching of the metal ion remains overall limited, supporting the major role of a surface-driven radical mechanism.

**Table 2:** Iron Release Detected by I.C.P.-O.E.S^a^

fiber	pH	time (d)	ppm Fe	mass Fe (mg)	mg Fe released/g fiber
CRO	7.4	1	<0.02	<0.00004	<0.004
CRO	7.4	3	0.069 ± 0.014	0.00014 ± 0.00002	0.014 ± 0.002
CRO	7.4	14	0.741 ± 0.074	0.00148 ± 0.00014	0.148 ± 0.014
CRO	4.1	1	2.79 ± 0.28	0.0056 ± 0.0006	0.557 ± 0.056
CHR	7.4	1	4.39 ± 0.22	0.00879 ± 0.00044	0.879 ± 0.088
CHR	7.4	3	10.73 ± 0.54	0.0215 ± 0.0011	2.15 ± 0.22
CHR	7.4	14	0.79 ± 0.04	0.00159 ± 0.00008	0.159 ± 0.016
CHR	4.1	1	6.72 ± 0.34	0.0134 ± 0.0007	1.345 ± 0.095
ERI	7.4	1	<0.02	<0.00004	<0.004
ERI	7.4	3	0.185 ± 0.009	0.00037 ± 0.00002	0.037 ± 0.004
ERI	7.4	14	0.452 ± 0.023	0.00090 ± 0.00005	0.090 ± 0.010
ERI	4.1	1	<0.02	<0.00004	<0.004

a Technique in solutions separated from suspensions made of CRO, CHR, and ERI and 3-CCA 10^–4^ M solutions at pH 7.4 after 1, 3, and 14 d and at pH 4.1 after 1 d.

At physiological pH (7.4), iron leaching from CRO and erionite ERI is limited across all investigated time points (1, 3, and 14 d), remaining low or even below the detection limit. As expected, under acidic conditions (pH 4.1), which mimic the intralysosomal environment, a slightly higher release of iron was observed after 1 d for the CRO. However, the absolute concentration of dissolved iron remained remarkably low, indicating that the bulk of the mineral framework remains intact and that acetate buffer does not cause extensive chelation-driven dissolution. Conversely, ERI exhibits a peculiar stability, maintaining values below the detection limit even at pH 4.1 after 24 h, further confirming the highly differentiated behavior of this zeolitic species compared to classic asbestos fibers.

In contrast, CHR exhibited a unique dynamic leaching profile. At pH 7.4, its iron release peaked after 3 d (2.15 mg of Fe/g fiber), a value significantly higher than that of the other two fibers. Intriguingly, this dissolved iron concentration sharply decreased after 14 d (0.159 mg of Fe/g fiber). This trend is highly consistent with an incongruent dissolution mechanism followed by the reprecipitation of dissolved iron back onto the fiber surface as highly insoluble secondary oxyhydroxide phases, a phenomenon well-documented under neutral or slightly acidic conditions.^31,32^ This surface precipitation process, which is likely followed by adsorption of 3-CCA and 7-OH-3-CCA, provides a possible explanation for the progressive drop in fluorescence and UV–vis signals observed over long incubation times for CHR at pH 7.4 (Figure 1). At pH 4.1, CHR exhibited a high release (1.345 mg Fe/g fiber after 1 d), driven by the expected acid-induced structural destabilization and magnesium leaching, which destabilizes the silicate framework.

Most importantly, radical production assays performed directly on the separated, fiber-free leachates by adding H_2_O_2_ to the collected supernatants confirm that the homogeneous Fenton contribution is minor, accounting for less than 10% of the total accumulated 7-OH-3-CCA signal (Supporting Information S8 and Figure S7a–c).

Taken together, the low concentration of mobilized iron across all conditions and the negligible radical activity detected in the leachates provide compelling, direct evidence that ^•^OH generation in our system remains overwhelmingly driven by a heterogeneous mechanism via active sites on the mineral surface, rather than by dissolved iron­(II)/iron­(III) eventually, at pH 4.1, also as acetate complexes.

Nevertheless, it is worth noting that iron leaching represents a key, slow pathological event that dynamically occurs in vivo within tissues following fiber inhalation. While surface-bound heterogeneous catalysis stands out as the primary and most immediate driver of oxidative stress, the progressive dissolution and mobilization of iron observed here contribute to the broader, long-term mechanism of toxicity and biopersistence of these mineral fibers.

### Production of Hydroxyl Radicals in an Acid Environment

According to what was observed at pH 7.4, at pH 4.1, the fluorescence spectra of the product formed by reacting the three fibers with 3-CAA and H_2_O_2_ show the spectrum of 7-OH-3-CAA at pH 4.1 (Supporting Information S2 and Figure S1). The calibration curve obtained at this pH (Supporting Information S3 and Figure S2b) was used to calculate the concentration of 7-OH-3-CAA. The results of the 3-CCA–H_2_O_2_–fluorescence method applied at different pH values on the same fibers, consecutively, after proper washing, are given in Table 3. The results are the mean values of two series of measurements, and the error is the maximum deviation.

**Table 3:** Values of Picomoles of 7-OH-3-CCA Produced by CRO, CHR, and ERI Following the 3-CCA–H_2_O_2_–Fluorescence Method Applied at Different pHs on the Same Fibers, Consecutively, after Proper Washing Procedures (See the Experimental Procedures Section)^a^

	pmol 7-OH-3CCA/mg pH 7.4 heterogeneous assay 48 h	pmol 7-OH-3-CCA/mg pH 7.4 for 48 h + pH 4.1 heterogeneous assay 24 h	pmol 7-OH-3-CCA/mg pH 4.1 for 24 h + pH 4.1 homogeneous assay 24 h	pmol 7-OH-3-CCA/mg pH 7.4 for 48 h + pH 4.1 for 24 h + pH 7.4 heterogeneous assay 5 h
CRO	8.8 ± 0.2	203 ± 1	42 ± 3	5.3 ± 0.1
CHR	5.2 ± 0.2	133 ± 9	17 ± 2	24 ± 4
ERI	2.4 ± 0.3	8 ± 1	0.5 ± 0.1	0.7 ± 0.2

a Column 3 reports the values of picomoles of 7-OH-3-CCA produced by leachate solutions separated from CRO, CHR, and ERI after 24 h in a 3-CCA solution at pH 4.1 and then measured with the 3-CCA–H_2_O_2_–fluorescence method after 24 h.

As shown in Table 3, a striking 20–25-fold increase in fluorescence and, indirectly, in ^•^OH production was observed for CRO and CHR at pH 4.1 compared to pH 7.4. To assess whether this massive amplification could be partially attributed to homogeneous Fenton chemistry driven by proton-induced fiber dissolution, specific control experiments were performed. Iron release was monitored via I.C.P.-O.E.S., and subsequent 7-OH-3-CCA production was measured in the separated leachates with the 3-CCA–H_2_O_2_–fluorescence method after exposing the fibers to a 10^–4^ M 3-CAA solution at pH 4.1 for 24 h. The results indicated that while acidic conditions lead to an increase in dissolved iron, the homogeneous Fenton reactivity in the filtered leachate accounts for less than 20% of the total radical yield, confirming that the process remains overwhelmingly surface-mediated (Table 3, third column).

However, this large increase in the fluorescence signal is not dictated by surface kinetics alone, but it is the net combination of two distinct factors:

1. The intrinsic fluorescence quantum yield of the 7-OH-3-CAA adduct is strongly pH-dependent. As evidenced by comparing the calibration curves established independently at pH 7.4 and pH 4.1 (Supporting Information S3 and Figure S2a,b), the slope at acidic pH is markedly steeper. The protonation of the carboxylic groups of 3-CCA intrinsically yields a higher emission intensity for the exact same concentration of 7-OH-3-CCA.
2. The increase of heterogeneous Fe^2+^ oxidation kinetics by H_2_O_2_ in acidic environments: The acidic environment promotes a partial proton-induced “etching” (microdissolution) of the fiber surface (see above). Probably, this process strips away unreactive outer layers and exposes a higher number of active iron sites, making them directly available to react with H_2_O_2_ through a highly efficient surface-mediated pathway.

### Understanding the Release of ^•^OH Radicals from the Three Mineral Fibers

Aware of the fact that our experiments were conducted in a simplified chemical system, hypotheses on the activity of the investigated fibers in the extracellular alveolar environment may help to understand the mechanism and explain the kinetics of iron-mediated release of primary ^•^OH from respirable mineral fibers. In fact, these ^•^OH radicals are responsible for the damage and alteration of the metabolism of cells of the respiratory tract, cytotoxicity, cell membrane lipid peroxidation, and other in vivo adverse effects.^33^

The generation of reactive oxygen species (ROS), namely, free radicals, represents a primary mechanism driving the cytotoxicity and genotoxicity of mineral fibers.^34,35^ In particular, the specific pathobiological response to crocidolite, chrysotile, and erionite is intimately linked to their surface reactivity and the chronic oxidative stress they induce in tissue microenvironments.^35,36^

When an iron-rich mineral fiber reaches the deep respiratory tract, there are basically three possible types of biochemical interaction:

(i) Extracellular detoxification of surface Fe^2+^ and Fe^3+^ (and eventually other metals) by chelating glycoproteins such as albumin (the most abundant), immunoglobulins, transferrin, lysozyme, the proteases cathepsin D, dipeptidyl peptidase 4, elastase, ascorbate, and more: These proteins are contained in the fluids of the alveolar gas exchange region and form a partial corona around the foreign particle;^37^
(ii) Extracellular activation of neutrophils and macrophages that, besides normal metabolic functions, release H_2_O_2_:^38^ This release produces primary ^•^OH through the Fenton-catalyzed reaction with available surface Fe^2+^ and, to a much lower extent, Fe^3+^.^10,11^ In fact, oxidation of Fe^2+^ by H_2_O_2_ is much faster than reduction of Fe^3+^,^39^ with the rate constant (in M^–1^ s^–1^) for the Fenton reaction at neutral pH Fe^2+^ + H_2_O_2_ → Fe^3+^ + ^•^OH + OH^–^ being at least 2 orders of magnitude greater than the Fenton reaction at neutral pH Fe^3+^ + H_2_O_2_ → Fe^2+^ + ^•^OOH + H^+^. Hence, Fe^2+^ is rapidly consumed, and the regeneration of Fe^2+^ via Fe^3+^ reduction becomes the rate-limiting step in sustained radical production;
(iii) Intracellular release of H_2_O_2_ by phagocytic cells, like alveolar macrophages, during the engulfment and clearance of the fibers. To dissolve the engulfed fiber, macrophages create phagolysosome sacs with an acidic environment and trigger an intense oxidative activity with the release of H_2_O_2_.^25,40^ The synergy of acidic pH and H_2_O_2_ production creates favorable conditions for a “burst” of ^•^OH radicals in contact with surface Fe^2+^ (and, possibly, Fe^3+^).^10,11^ During this process, phagocytic cells also release secondary ^•^OH.^25^

In our experiments, the extracellular environment at physiological conditions was simulated with the addition of the spot tests at acidic pH, mimicking the situation of intracellular phagolysosome engulfment (pH = 4.1) in contact with a fiber. All the fibers were structurally stable during the course of the experiments as shown by the XRPD traces collected on the solid fibers separated from the solutions containing the reaction products of CRO, CHR, and ERI at pH 7.4 after 48, 264, and 720 h (792 h for ERI) (see the XRPD patterns after 720 h for CHR and CRO and 792 h for ERI, respectively, in the Supporting Information S9 and Figure S8).

It was observed that the mechanism of iron-mediated release of primary ^•^OH radicals is different for the three fibers CRO, CHR, and ERI.

Crocidolite (CRO) is an iron-rich amphibole asbestos with octahedrally coordinated ferrous and ferric iron (Fe^2+^_2.34_ and Fe^3+^_2.05_ in the unit formula, corresponding to 14.3 wt % Fe^2+^ and 12.6 wt % Fe^3+^, respectively). The curve of ^•^OH released from CRO (Figure 1) reaches a plateau after about 72 h, in perfect agreement with that drawn by Andreozzi et al.^41^ for the total [DMPO–OH]• production of the UICC crocidolite standard during leaching at pH 7.4. From a mechanistic viewpoint, this long-term radical generation is proposed to follow a multistep kinetic profile driven by the different reaction rates of surface iron species. Production of ^•^OH is initially due to the activation of catalytic Fe^2+^ surface sites, which are quickly consumed in a rapid oxidation step. Fe^3+^ surface sites are also reduced by H_2_O_2_ to active catalytic Fe^2+^ sites, but this reaction is 2 orders of magnitude slower than Fe^2+^ oxidation, acting as the rate-limiting step for the production of ^•^OH over longer time scales.

Control experiments on separated leachates confirmed that at pH 7.4, the homogeneous Fenton contribution from dissolved iron is minor, accounting for less than 10% of the total accumulated signal, verifying that radical generation remains predominantly driven by surface-active sites under neutral conditions.

Partial dissolution of the nanometric surface layer of the fiber observed by TEM elsewhere^42^ prompts migration of iron atoms from the bulk to the fiber surface, aggregating into newly formed Fe^3+^-rich oxide–hydroxide nanoparticles. Further production of ^•^OH from these Fe^3+^-rich nanoparticles is again limited by the very slow reaction of reduction of the Fe^3+^ species.

Although our experiments were conducted in a simplified chemical system, it is possible to expect that a respirable crocidolite fiber reaching the extracellular alveolar environment at physiological pH is active in producing primary ^•^OH from the rapid surface Fe^2+^ oxidation (see Figure 1). As the reaction progresses (*t* > 120 h), the kinetic profile evolves toward a slower generation of ^•^OH sustained by the continuous redox cycling of surface Fe^3+^ reacting with H_2_O_2_ via a Haber–Weiss/Fenton-like reaction.^41^ This multistep kinetic behavior represents an empirical interpretation of our data under these experimental conditions, aligned with the known behavior of mixed-valence iron minerals. The curve of CRO in Figure 1 reaches a plateau because the H_2_O_2_ pool is depleted. This is supported by the “refill” point (red ▲) at 72 h present in Figure 1 that represents new ^•^OH radical production from the fiber in contact with a fresh H_2_O_2_-rich solution.

Chrysotile (CHR) is a layer silicate with a cylindrical lattice composed of Si^4+^-centered tetrahedral sheets alternating with Mg-centered octahedral sheets. Ferrous iron (Fe^2+^_0.027_ in the unit formula, corresponding to 0.54 wt % Fe^2+^) replaces magnesium in the octahedral cavities,^43^ while ferric iron (Fe^3+^_0.044_ in the unit formula, corresponding to 0.88 wt % Fe^3+^) occupies both octahedral and tetrahedral sites. The curve of the ^•^OH radical released from CHR (Figure 1) increases with a shape similar to that of CRO, but after 48 h, it anomalously decreases. As supported by I.C.P.-O.E.S. control assays, this decline is triggered by an incongruent dissolution mechanism characteristic of chrysotile in neutral or slightly acid solutions: the outermost magnesium layers rapidly dissolve, inducing the subsequent reprecipitation of leached iron back onto the fiber surface as highly insoluble secondary oxyhydroxide phases.^31,32^ This dynamic surface restructuring leads to the progressive adsorption of both the 3-CCA probe and its 7-OH-3-CCA fluorescent adduct on the high specific surface area of CHR fibers, resulting in the observed attenuation of the solution signal (see above and Figure 2). Independent leachate tests (Supporting Information S8 and Figure S7a–c) established that at neutral pH, the true homogeneous Fenton pathway is minimal.

Despite the low iron content with respect to CRO, the production of primary ^•^OH from CHR is relevant because, in addition to the Fenton-catalytic Fe^2+^ sites present on the fiber surface, new surface sites develop due to the migration of Fe^2+^ ions from the inner Mg^2+^-rich octahedral sheet to the fiber surface. Mg^2+^ and Fe^2+^ diffusion toward the fiber surface at neutral pH is commonly described as a layer-by-layer dissolution. Mg^2+^-rich sheets on the fiber surface dissolve within hours, whereas exposed Si^4+^-rich sheets dissolve much more slowly and therefore control the overall dissolution rate. Fe^2+^ remains Fenton-active during long-term dissolution (weeks) because the Si^4+^ sheet in which it is incorporated dissolves slowly.^19,44,45^ Newly formed Fe^3+^ surface sites are possibly reduced later by H_2_O_2_ to active catalytic Fe^2+^ sites, but this reaction is 2 orders of magnitude slower than Fe^2+^ oxidation, which remains the dominant initial reaction for the production of ^•^OH.

The curve fit shown in red in Figure 1 suggests that a respirable chrysotile fiber in the extracellular alveolar environment at physiological pH is also active, like crocidolite, in producing primary ^•^OH from surface Fe^2+^ oxidation. As for the CRO case, a minor continuous amount of ^•^OH can also be produced over time by the slower reduction of surface Fe^3+^ to active Fe^2+^.^19,32^ The kinetic curve reaches a plateau also for CHR because the H_2_O_2_ pool is depleted as demonstrated by the “refill” point (red ▲) at 72 h in Figure 1 following fiber contact with a fresh H_2_O_2_-rich solution.

During the 168 h pretreatment of the refill test executed in the absence of the probe, the fiber undergoes surface evolution and proton-induced etching without the immediate competitive masking or complexation by the organic molecule. This pristine surface evolution enhances the accessibility of reactive iron sites, explaining why CHR displays a 2-fold increase in ^•^OH production upon reintroduction of the fresh probe mixture.

Erionite (ERI) is a fibrous zeolite composed of a framework silicate with Si^4+^ and Al^3+^ at the center of connected tetrahedra forming the 3-dimensional framework, along with extraframework cations surrounded by water molecules in the micropores inside the framework. Iron associated with erionite is 0.59 wt % Fe^3+^, but it does not belong to the zeolite structure. It is hexa-coordinate Fe^3+^ ions contained in different iron-bearing impurities (amorphous iron-rich nanoparticles, microparticles of iron oxides/hydroxides, and flakes of nontronite) that occur at the surface of the erionite fibers.^46^ The curve of ^•^OH released from ERI (Figure 1) has a different shape with respect to CRO and CHR. The reaction rate is an order of magnitude slower than the other fibers (Table 1), and the curve reaches a plateau after about 800 h. The reaction is slower because ^•^OH are produced by the Fe^3+^ from the nanoimpurities at the fiber surface that must be reduced by H_2_O_2_ to generate active catalytic Fe^2+^ sites. This process has already been well described in the literature for iron hydroxides like goethite (FeOOH). Moreover, it was shown that the prevailing pathway of ^•^OH generation was due to the decomposition of H_2_O_2_ catalyzed by the active sites on the goethite surface.^47^ As shown above, this reaction is found to be 2 orders of magnitude slower than Fe^2+^ oxidation. The reaction rate observed here (Table 1) is actually only 1 order of magnitude slower for ERI with respect to CRO and CHR. There are two possible reasons to explain the increase of the kinetic rate in the ERI sample: (i) the kinetic rate is increased by the nanophasic nature of the iron-rich impurities with a large active specific surface; and (ii) as reported in Gualtieri et al.,^46^ a fraction of iron can be found as dispersed isolated iron species anchored at the erionite framework surface or as low nuclearity species at the interface between the iron nanophase and the erionite framework surface. In both cases, ^•^OH production tends to be faster when Fe^3+^ is closely associated with Brønsted acid sites (like framework Al atoms) in zeolites, especially under neutral pH conditions. In fact, due to nearby Brønsted acid sites, the local environment around the Fe^3+^ can be slightly acidic, thus mimicking the acidic pH, which speeds up the Fenton reaction, improving H_2_O_2_ activation and Fe^3+^ reduction.^48–50^

Although our experiments were conducted in a simplified acellular system, our findings suggest that erionite fibers reaching the extracellular alveolar environment at physiological pH are expected to generate primary ^•^OH radicals over an extended period (Figure 1). These ^•^OH radicals are predominantly produced by derivative Fe^2+^ ions due to the slow reduction of surface Fe^3+^ from the nanophasic impurities or dispersed species at the surface of the erionite fibers.

It was observed that the production of ^•^OH radicals after 2 h (Figure 1) decreases in the following order: *I*_em,CRO_ > *I*_em,CHR_ > *I*_em,ERI_. This result is consistent with the intracellular ROS production reported by Mirata et al.^51^ in different macrophage phenotypes (M0, M1, and M2; see Figure 6 in Mirata et al. 2022), following 2 h exposure to the same CHR, CRO, and ERI fibers. Their data show that, in all differentiated macrophage types, the three mineral fibers significantly increased intracellular ROS levels compared with untreated control cells, with the trend ROS_CRO_ > ROS_CHR_ > ROS_ERI_. We suggest that the ROS observed by Mirata et al.^51^ primarily originate from iron present on the fiber surfaces.

Considering the different amount of iron, and in particular of Fe^2+^, present in the three fibers (very high in CRO with 14.3 wt % Fe^2+^ and 12.6 wt % Fe^3+^; very low in CHR, with 0.54 wt % Fe^2+^ and 0.88 wt % Fe^3+^; and in ERI, with 0.59 wt % Fe^3+^), the values of 7-OH-3-CCA picomoles produced for mass unit of fiber derived from Figure 1 are apparently inconsistent. In fact, CRO is expected to display much higher values than the other fibers. However, this is not the case because high catalytic activity and production of ^•^OH are observed when iron atoms are isolated species, namely, when iron atoms display nuclearity (*n*, the number of iron atoms connected by one bridging oxygen atom) *n* = 1. On the other hand, catalytic activity is expected to be significantly reduced when iron forms clusters of higher nuclearity, as observed in crocidolite.^24^

The experiments conducted at pH 4.1 after 48 h at pH 7.4 (Table 2) were planned to mimic the case of fiber engulfment by alveolar phagocytes and subsequent contact with intracellular acidic phagolysosomes,^20,28^ an event that may occur after 2 d of residence in the alveolar extracellular space.

I.C.P.-O.E.S. controls performed under acidic conditions confirmed that proton-induced dissolution increases, releasing iron into the solution (Table 2). While the homogeneous Fenton pathway contributes a minor fraction to the overall yield at early points (accounting for less than 10% of the initial radical yield in short-term leachate tests), its role represents a parallel, complementary pathway under prolonged acidic incubation conditions (Table 3). This indicates that at pH 4.1, the observed radical generation is a combined outcome of the dominant surface-mediated mechanics and a mobile homogeneous Fenton contribution from dissolved iron.

The results confirm that mild acid pH in the presence of H_2_O_2_ induces a burst of ^•^OH radicals in contact with iron.^10,11^ In fact, the number of 7-OH-3-CCA picomoles per mass unit of fiber produced at pH 4.1 is 23 times larger than that measured at physiological pH for CRO, 26 times for CHR, and 3 times for ERI (Table 3).

This massive fluorescence signal amplification observed for CRO and CHR cannot be solely ascribed to the surface kinetics. As highlighted by our independent calibration curves (Supporting Information S3), the intrinsic fluorescence quantum yield of the 7-OH-3-CCA is strongly pH-dependent; the protonation of the coumarin derivative under acidic conditions inherently provides a significantly higher emission intensity for the same nominal amount of radical adduct. Therefore, the 23- to 26-fold spike is the combined result of a dominant photophysical enhancement and a secondary chemical acceleration. The chemical contribution is driven by the known difference in the heterogeneous rate constant of Fe^2+^ oxidation by H_2_O_2_ (e.g., goethite), which increases from 0.0225 M^–1^ s^–1^ at pH = 7 ^52^ to 1.22 M^–1^ s^–1^ at pH = 4.^53^

Instead, the slight variation in 7-OH-3-CCA production at the two pHs observed for ERI, whose ^•^OH production is governed by Fe^3+^ reduction, is explained by the minor difference in the rate constant of Fe^3+^ reduction by H_2_O_2_ with pH in heterogeneous systems: 2.3 × 10^–5^ M^–1^ s^–1^ at pH = 4 *vs* 1.1 × 10^–5^ M^–1^ s^–1^ at pH = 7.^54^ The fourth column of Table 3 shows that after the burst of ^•^OH following the simulation of the phagolysosome engulfment at pH 4.1, all fibers continue to produce amounts of radicals comparable to those observed before the event.

Our experiments reproduced ideal conditions of a fiber in the extracellular environment at physiological pH for about 720 h with spikes of acidic conditions mimicking the case of intracellular phagolysosome engulfment during the tentative clearance by alveolar phagocytes. Although our experiments are only simplified chemical systems, some speculations about the fate of the fibers in vivo in the long run can be made.

Crocidolite is a biodurable fiber^55^ that resists well in the acidic phagolysosome intracellular environment. For long residence times in the extracellular space, contrary to what is observed at short times, when Fe^2+^ at the fiber surface is the dominant iron species responsible for the ^•^OH burst, the continuous radical generation can be attributed to the progressive reduction cycle of surface Fe^3+^. As a proposed interpretation of our long-term kinetic curves, the primary pool of pristine surface Fe^2+^ undergoes rapid depletion, leaving the slower reduction of Fe^3+^ as the mechanism that recharges the catalytic cycle, reacting with H_2_O_2_ to produce Fe^2+^ according to the reaction: Fe^3+^ + H_2_O_2_ → Fe^2+^ + O_2_^•–^ + 2 H^+^. To a much lesser extent, Fe^2+^ may eventually migrate to the surface from the bulk of the fiber and react with H_2_O_2_. Accordingly, some studies explicitly support the “restart/redox-cycling” model where surface Fe^2+^ is first oxidized to Fe^3+^ and later reduced back with a slower kinetics to Fe^2+^, thereby enabling renewed ^•^OH production via the Haber–Weiss reaction.^19,41^ Therefore, the observed sequential behavior represents a specific empirical finding of this study under the tested conditions, where the initial ^•^OH burst is governed by primary Fe^2+^ oxidation, whereas the long-term process is driven by the slower, rate-limiting Fe^3+^ surface reduction loop.

The H_2_O_2_ needed for the activation of iron is eventually released by neutrophils and macrophages^38^ or formed during normal extracellular metabolic functions. Intracellular spikes of ^•^OH radicals are produced during acidic phagolysosome engulfment whenever alveolar phagocytosis occurs. In that case, migration of catalytically active Fe^2+^ species from the bulk to the surface of the fiber is also favored.

Chrysotile is nonbiodurable in an acid environment;^57^ a fiber of 0.25 μm thickness dissolves in a time span of 2000–4000 h (94–177 d).^56^

In the extracellular space, ^•^OH will also be chronically produced but to a lower extent with respect to crocidolite, following the reduction cycle of residual surface Fe^3+^ (again, the predominant iron species left at the surface of the fiber after the depletion of the initial Fe^2+^ pool) in contact with H_2_O_2_.

Erionite is another acid-resistant biodurable fiber.^46^ Apparently, the behavior in the long run is comparable to that of crocidolite with some exceptions: (i) natural erionite is virtually Fe^2+^-free; (ii) production of ^•^OH radicals from the reduction cycle of surface Fe^3+^ is favored by the more reactive nanophasic iron-rich impurities; and (iii) a lower intensity of ^•^OH production occurs during the phagocytosis acid spikes.

Finally, it should be noted that while our cell-free model strictly quantifies the primary, iron-mediated pathways of radical generation, “secondary” ^•^OH radicals (not investigated here) will be chronically produced in vivo by inflammatory cells. This occurs because long, high-aspect-ratio fibers deeply alter macrophage functions; even when incomplete engulfment prevents clearance, the continuous fiber–cell interaction activates sustained intracellular signaling.^57^

## Conclusions

This work delivers a novel, highly sensitive spectrofluorimetric procedure exploiting 3-coumarin carboxylic acid (3-CCA) as a molecular probe to systematically track and compare the generation kinetics of hydroxyl radicals ^•^OH produced by the carcinogenic mineral fibers crocidolite, chrysotile, and erionite. A key advantage of this methodology is its capability to capture the early accumulation profiles of primary ^•^OH before they are effectively consumed by complex secondary surface reactions. Furthermore, by utilizing tailored calibration curves under identical, strictly controlled experimental conditions, the comparative yield of radical production over time per mass unit of fiber can be reliably evaluated within a highly consistent framework.

When evaluating radical generation by hazardous mineral fibers, electron paramagnetic resonance (EPR) spectroscopy coupled with spin trapping represents the definitive tool for the unambiguous identification and absolute quantification of specific radical species. Nevertheless, EPR spin trapping can be synthetically constrained by the limited chemical half-life of the radical adducts during prolonged incubations. In this context, the steady-state fluorimetric approach using coumarin-3-carboxylic acid offers a robust and innovative kinetic framework. Because the resulting fluorescent product is highly stable over time, this method allows for the continuous, long-term monitoring of ^•^OH release kinetics. Our findings demonstrate that these mineral interfaces do not act as passive, short-lived triggers; instead, they sustain and dynamically renew their radical-generating capacity over extended periods through distinct structural mechanisms:

- Crocidolite and erionite exhibit remarkably different long-term kinetic profiles. This divergence is fundamentally dictated by the nature of the iron active sites on their surfaces, specifically the oxidation states (the dominance of highly reactive structural Fe^2+^ clusters in crocidolite versus the slower, rate-limiting reduction of structural Fe^3+^ nanoimpurities in erionite) and differences in site nuclearity. Our model proposes a multistep kinetic behavior for crocidolite as an empirical interpretation of its long-term recharge capacity.
- Chrysotile, despite its low iron content, exhibits a significant initial radical generation capacity. Its kinetic profile highlights a complex surface evolution governed by incongruent magnesium dissolution and subsequent iron oxyhydroxide reprecipitation, which drives the progressive masking of active sites and cross-complexation of the organic probe in circumneutral environments.

Finally, by successfully simulating the transition from extracellular physiological pH to the acidic conditions (pH 4.1) of the intracellular phagolysosome, this model successfully reproduces the combined analytical and chemical “burst” of radical tracking expected during macrophage engulfment while accounting for the complementary, parallel contribution of iron dissolution and homogeneous Fenton chemistry under prolonged acidic incubation.

## Supplementary Material



## Notes

Characterization of fibers and chemical reagents; fluorescence spectra of 7-OH-3-CCA at pH 7.4 and at pH 4.1; calibration curves of 7-OH-3-CCA at pH 7.4 and at pH 4.1; instrumental techniques; fluorescence spectra of the solutions separated from CRO, CHR, and ERI at different times; time course of the intensity of the fluorescence emission of the 7-OH-3-CCA species produced by fibers without and with TEMPO scavenger; UV–vis spectra of the solutions separated from CRO and ERI at different times; 3-CCA–H_2_O_2_–fluorescence method applied to leachate solutions to detect homogeneous Fenton contribution; X-ray powder diffraction (XRPD); and references (PDF)
