# Supplementary material for: Kinetics of H2O2‑Induced Hydroxyl Radical Release by Carcinogenic Mineral Fibers Crocidolite, Chrysotile, and Erionite
Source: Chem Res Toxicol. 2026 Aug 24;39(9):1659–71. doi: 10.1021/acs.chemrestox.6c00003 (PMC13599473; doi:10.1021/acs.chemrestox.6c00003)
Supplement: Supplementary file 1 [file tx6c00003_si_001.pdf]

Supporting Information

Kinetics of H_2O_2-Induced Hydroxyl Radical Release by Carcinogenic Mineral Fibers Crocidolite, Chrysotile, and Erionite

Elena Castellini* and Alessandro F. Gualtieri

Department of Chemical and Geological Sciences, University of Modena and Reggio Emilia, 41125, Modena, Italy.

*Corresponding author:
Elena CASTELLINI, PhD
Department of Chemical and Geological Sciences
University of Modena and Reggio Emilia
Via Campi, 103
41125 MODENA
ITALY
Tel: 0039-059-2058679
e-mail: elena.castellini@unimore.it

Table of contents: S1: Characterization of fibers and chemical reagents (page 2); S2: Fluorescence spectra of 7-OH-3-CCA at pH 7.4 and at pH 4.1 (page 4); S3: Calibration curves of 7-OH-3-CCA at pH 7.4 and at pH 4.1 (page 4); S4: Instrumental techniques (page 6); S5: fluorescence spectra of the solutions separated from CRO, CHR and ERI at different times (page 8); S6: Time course of the intensity of the fluorescence emission of the 7-OH-3-CCA species produced by fibers without and with TEMPO scavenger (page 10); S7: UV-Vis spectra of the solutions separated from CRO and ERI at different times (page 11); S8: 3-CCA-H_2O_2-fluorescence method applied to leachate solutions to detect homogeneous Fenton contribution (page 12); S9: X-ray powder diffraction (XRPD) (page 15); References (page 16).

S1

Mineral fibers and chemical reagents

The mineral fibers analysed in this study are crocidolite (CRO), chrysotile (CHR) and erionite (ERI). Although all of them share common characteristics like being fibrous natural silicates classified by the International Agency for Research on Cancer (IARC) in *Group 1* as "carcinogens (mainly mesothelioma) for humans",[1] their crystal-chemical assembly is very different.[2] The amphibole asbestos crocidolite is a double-chain silicate with ideal chemical formula $Na_2(Fe^{2+},Mg)_3Fe_2^{3+}Si_8O_{22}(OH)_2$. Chrysotile is a layer silicate with a trioctahedral hydrous layered structure composed of a Si-centered tetrahedral sheet (T) and a Mg-centered octahedral sheet (O). The ideal chemical formula is $Mg_3(OH)_4Si_2O_5$. Erionite is a framework silicate with mean ideal formula $K_2(Na,Ca_{0.5})_7[Si_{27}Al_9O_{72}]$ $28H_2O$. It is a widespread natural zeolite whose structure is composed of columns of cancrinite cages connected by a double six-membered ring of tetrahedra, forming hexagonal prisms. The samples investigated in this study are:

-a representative sample of UICC crocidolite standard (South African, NB #4173–111–3), named CRO hereafter. The sample contains minor (<1 wt%) hematite, magnetite, and quartz. The chemical

formula is $(Na_{1.96}Ca_{0.03}K_{0.01})_2(Fe^{2+}_{2.34}Fe^{3+}_{2.05}Mg_{0.52})_{4.91}(Si_{7.84}Al_{0.02})_{7.86}O_{21.36}(OH)_{2.64}$. This sample has mean length of 18.0 μm, mean width of 0.35 μm and specific surface specific area of 16.1 m^2/g.[3]

-a commercial chrysotile sample (CHR hereafter) from the Orenburg Minerals mine near Yasny, southern Ural Mountains (Russia). The sample is composed of clinochrysotile with minor orthochrysotile, and possibly very minor impurities of lizardite-1T, magnetite, hydromagnesite, and calcite. The chemical formula is $(Mg_{2.870}Fe^{2+}_{0.027}\ Fe^{3+}_{0.044}Al_{0.034}Cr_{0.005}Ni_{0.006})_{2.986}(OH)_4Si_{1.982}O_5$. This sample has mean length of 29.8 μm, mean fiber width of 0.4 μm, and specific surface area of 28.9(1.2) m^2/g.[3]

-erionite from Jersey, Nevada (USA) (ERI hereafter). This sample is pure, with chemical formula: $(Na_{5.31}K_{2.18}Ca_{0.15}Mg_{0.11}Fe^{3+}_{0.29})_{8.04}(Si_{27.84}Al_{7.85})_{35.69}O_{72}\cdot 20.3H_2O$ Iron ($Fe^{3+}_{0.29}$ is present as nanoparticulate impurities at the fiber surface). This sample has mean length of 16 μm, mean fiber width of 0.5 μm, and specific surface area of 12.7 m^2/g.[4]

The following chemicals were used: coumarin-3-carboxylic acid 99% (3-CCA, hereafter), and H_2O_2 solution 34.5-36.5%, both from Sigma Aldrich; dihydrogen sodium phosphate (NaH_2PO_4), disodium hydrogen phosphate (Na_2HPO_4) sodium acetate trihydrate (NaAc x $3H_2O$) salts, and acetic acid glacial (HAc) all RPE from Carlo Erba; 7-hydroxycoumarin-3-carboxylic acid, ≥ 98.0%, (7-OH-3-CCA, hereafter) from Merck; 2,2,6,6-tetramethylpiperidin-1-yl)oxyl (TEMPO) 98% from Sigma Aldrich.

S2

Fluorescence spectra of 7-OH-3-CCA

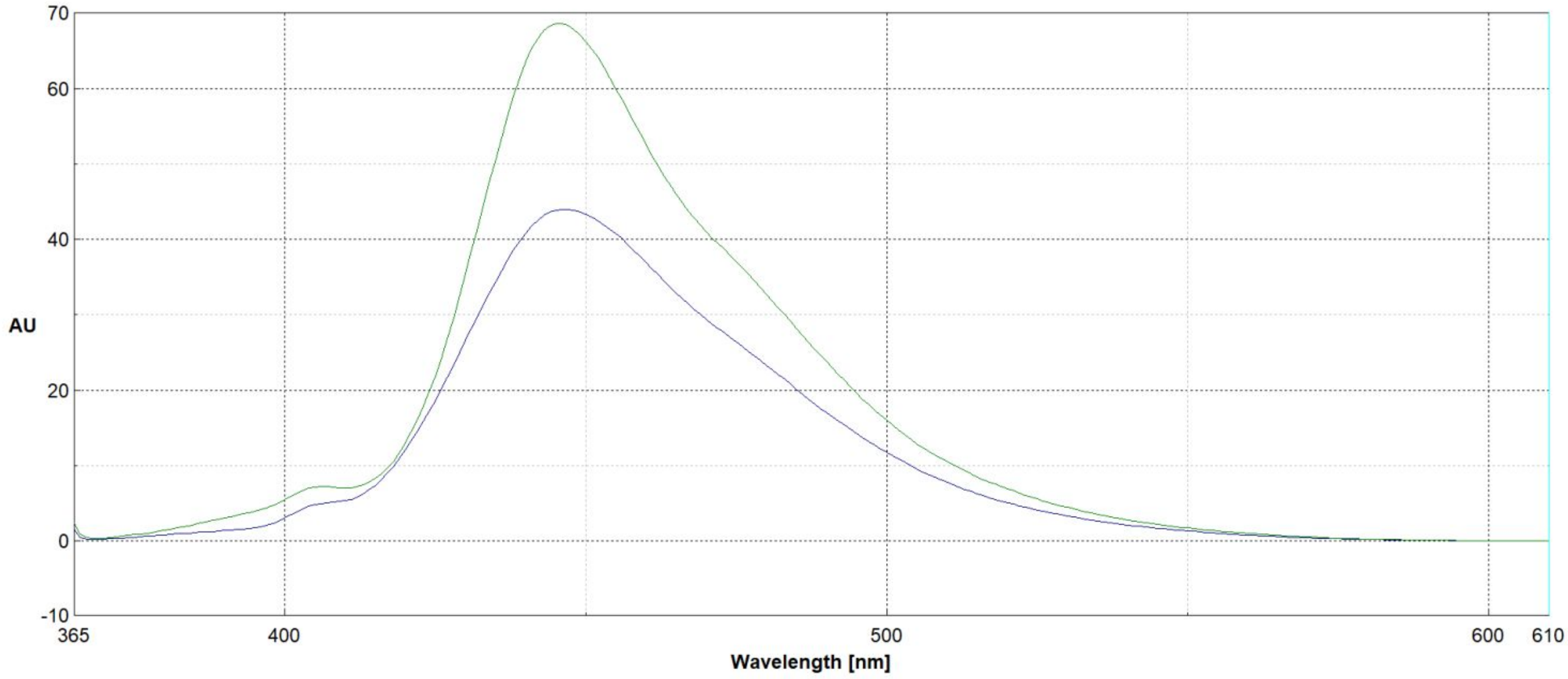

Figure S1. Fluorescence emission spectra of 7-OH-3-CCA at concentration 0.09 µM at pH 7.4 (blue) and at pH 4.1 (green).

S3

Calibration curves of 7-OH-3-CCA

To measure the production of the hydroxyl radicals by the fibers, the calibration curves reporting the fluorescence emission intensity of the signal at 446 nm in the spectrum of 7-OH-3-CCA as a function of its concentrations were performed at pH 7.4 (**Figure S2a**) and 4.1 (**Figure S2b**). The pH 7.4 was obtained with phosphate buffer, while the pH 4.1 with acetate buffer. For each concentration of 7-OH-3-CCA, fluorescence measurements were repeated at least three times and the corresponding equation calculated using all the points with *Sigma Plot version 12.3*.

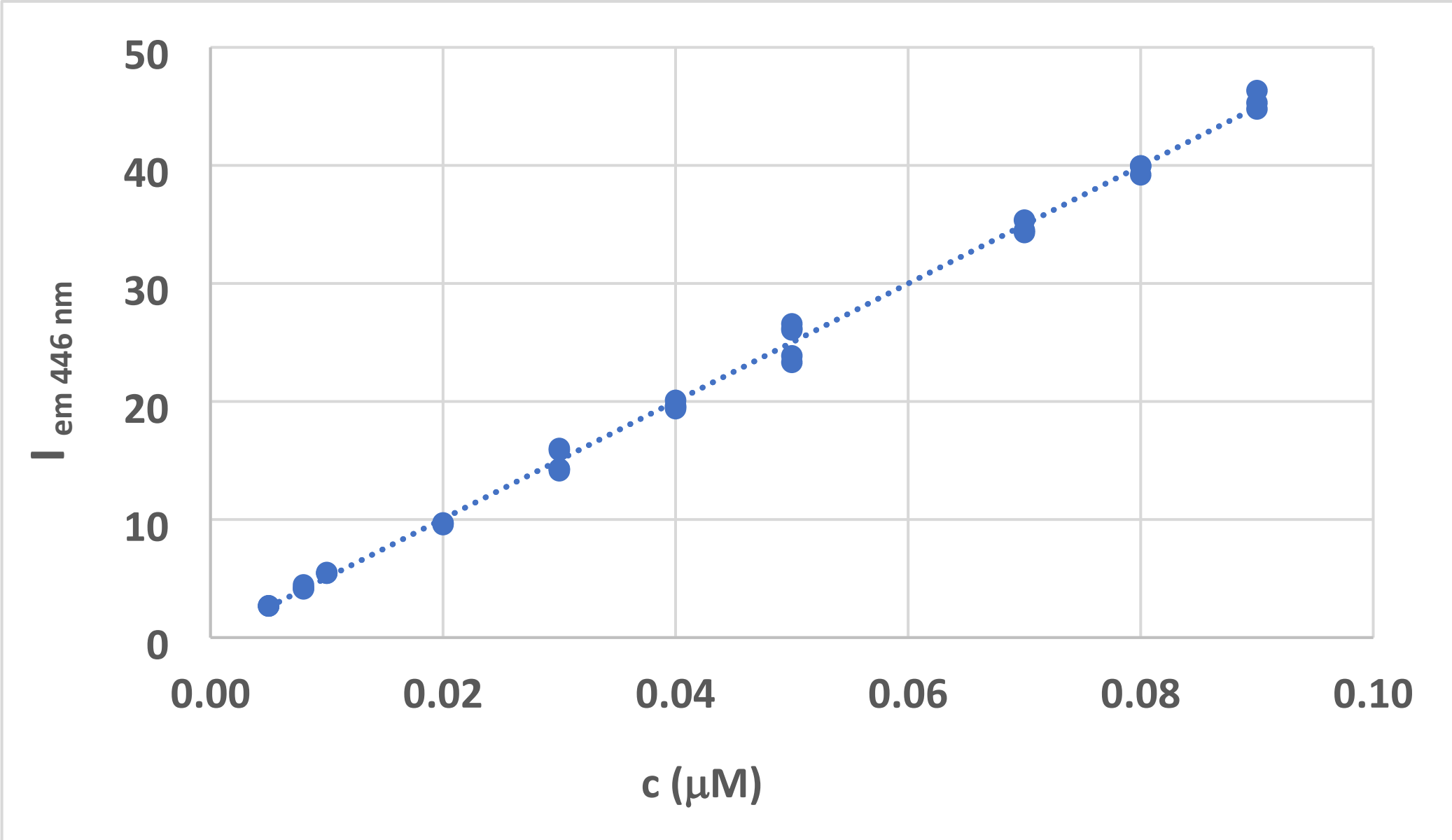

(a)

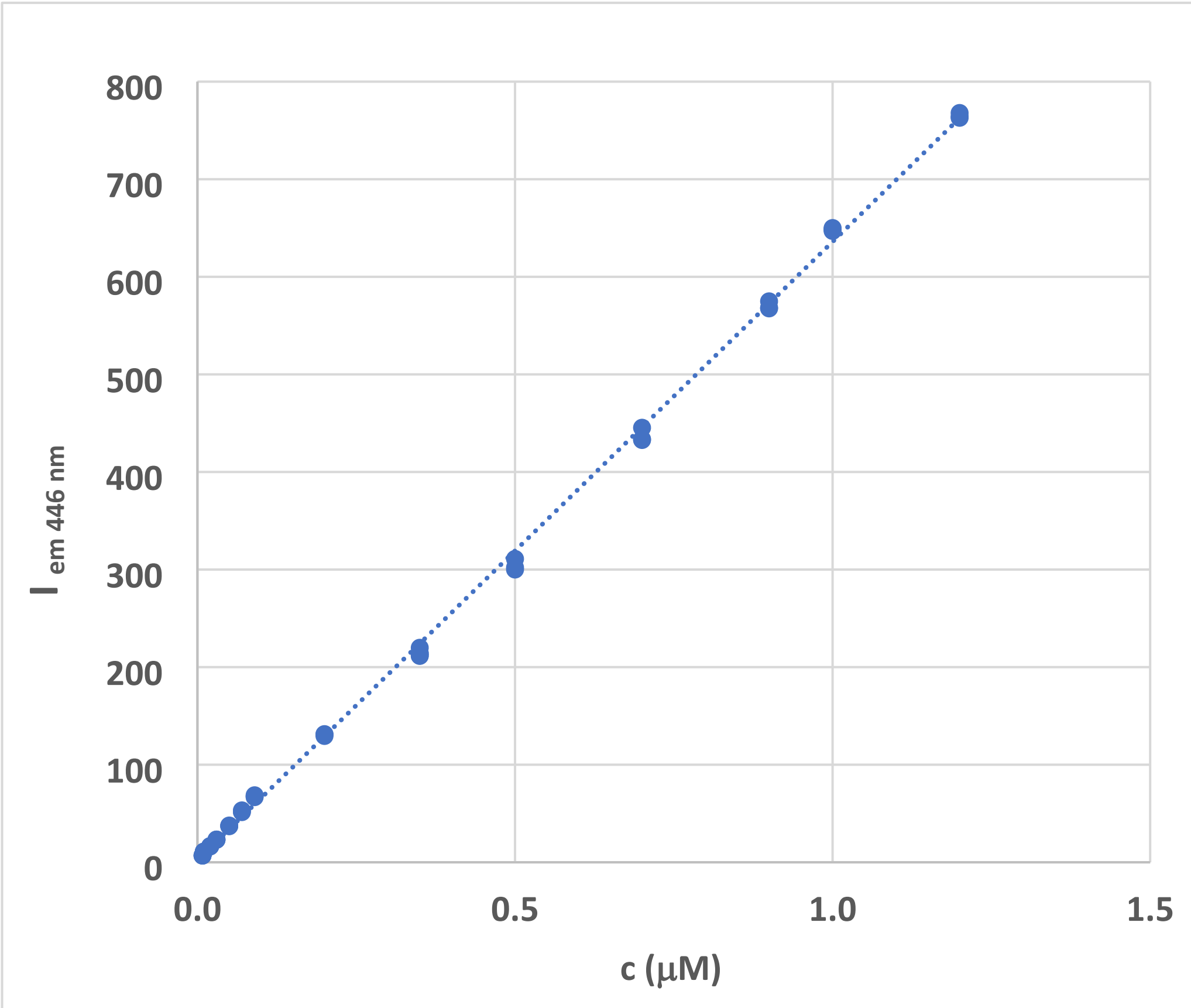

(b)

Figure S2. Fluorescence emission intensity of the signal at 446 nm in the spectrum of 7-OH-coumarin-3-CCA at pH 7.4 (10 mM phosphate buffer) as a function of its concentrations (a); fluorescence emission intensity of the signal at 446 nm in the spectrum of 7-OH-coumarin-3-CCA at pH 4.1 (10 mM acetate buffer) as a function of its concentrations (b).

The best fitting of each calibration curve provided a linear equation of the type:

$I_{emission\ 446\ nm} = a * c + b$ (I)

where $I_{emission\ 446\ nm}$ is the fluorescence emission intensity of the signal at 446 nm, c is the concentration of 7-OH-coumarin-3-CCA, while *a* and *b* are slope and intercept of the straight line, respectively. The *a* and *b* parameters are reported in Table S1 with the corresponding square correlation coefficients for the two calibration curves.

Table S1. Values of *a* and *b* parameters of the equation (I) obtained by the best fitting of the data of the calibration curves at pH 7.4 and 4 (**Figures S2a and S2b**) with *Sigma Plot version 12.3.*

	a **(μM^{-1})**	***b***	**R^2**
**pH 7.4**	498 ± 4	0.09 ± 0.21	0.9974
**pH 4.1**	633 ± 3	2 ± 2	0.9991

Using these parameters of the calibration curves it was possible to convert the data of fluorescence emission intensity in the corresponding concentration of 7-OH-3-CAA, which is a proxy of the amount of radical hydroxyls produced by reaction of the 3-CCA and H_2O_2 in the presence of the fibers.

S4

Instrumental techniques

Fluorescence measurements were performed with a Jasco instrument (FP-6200 model), employing the following conditions: excitation wavelength: 355 nm; emission wavelength range: 365-610 nm. The excitation wavelength of 355 nm was chosen on the basis of a set of preliminary measurements performed with a number of excitation wavelengths (332, 365, 395 nm), which were selected on the basis of the UV-Vis spectrum of 3-CCA (**Figure S3**). The fluorescence spectra for all the supernatants separated from the three fibers at different times (as described above) were recorded. The final

fluorescence spectra were obtained subtracting to each spectrum the blank spectrum recorded immediately after the addition of 20 μl of the 10^{-2} M H_2O_2 solution to 1 ml of the 3-CCA sample.

UV-Vis measurements (Jasco, V-570 model) were performed on the starting 10^{-4} M 3-CCA solution and on the same solutions immediately after the fluorescence analysis to check if adsorption of the 7-OH-3-CCA fluorescent product occurs which may affect its detection by fluorescence.

X-ray powder diffraction (XRPD) was used to study the samples of solid fibers separated from the solutions containing the reaction products of CHR, CRO and ERI after 48, 264 and 720 h (792 h for ERI). XRPD patterns were collected using a θ–θ Bragg-Brentano PANalytical *'XPert Pro* Diffractometer equipped with a Cu Kα radiation (λ=1.5418 Å) tube operating at 40 kV and 30 mA and with a real time multiple strip (RTMS) detector. The samples were ground using an agate mortar and the powder was mounted on a zero-background Si holder. Data were collected in the angular range 3–15 °2θ, using a step scan of 0.0167 °2θ and a counting rate of 100-200 s/step. A 0.5° divergence slit was used together with 0.04 rad Soller slits. The XRPD patterns were analysed using the *X-Pert High Score Plus* suite.[5]

I.C.P.-O.E.S. (Inductively Coupled Plasma - Optical Emission Spectroscopy) technique (I.C.P.-O.E.S. 4200 DV Perkin Elmer) was applied to determine iron concentration in the leachate solutions separated from CRO, CHR and ERI after 1, 3 and 14 days of contact with 10^{-4} M 3-CCA solution at pH 7.4 and 4.1 (T=37°C).

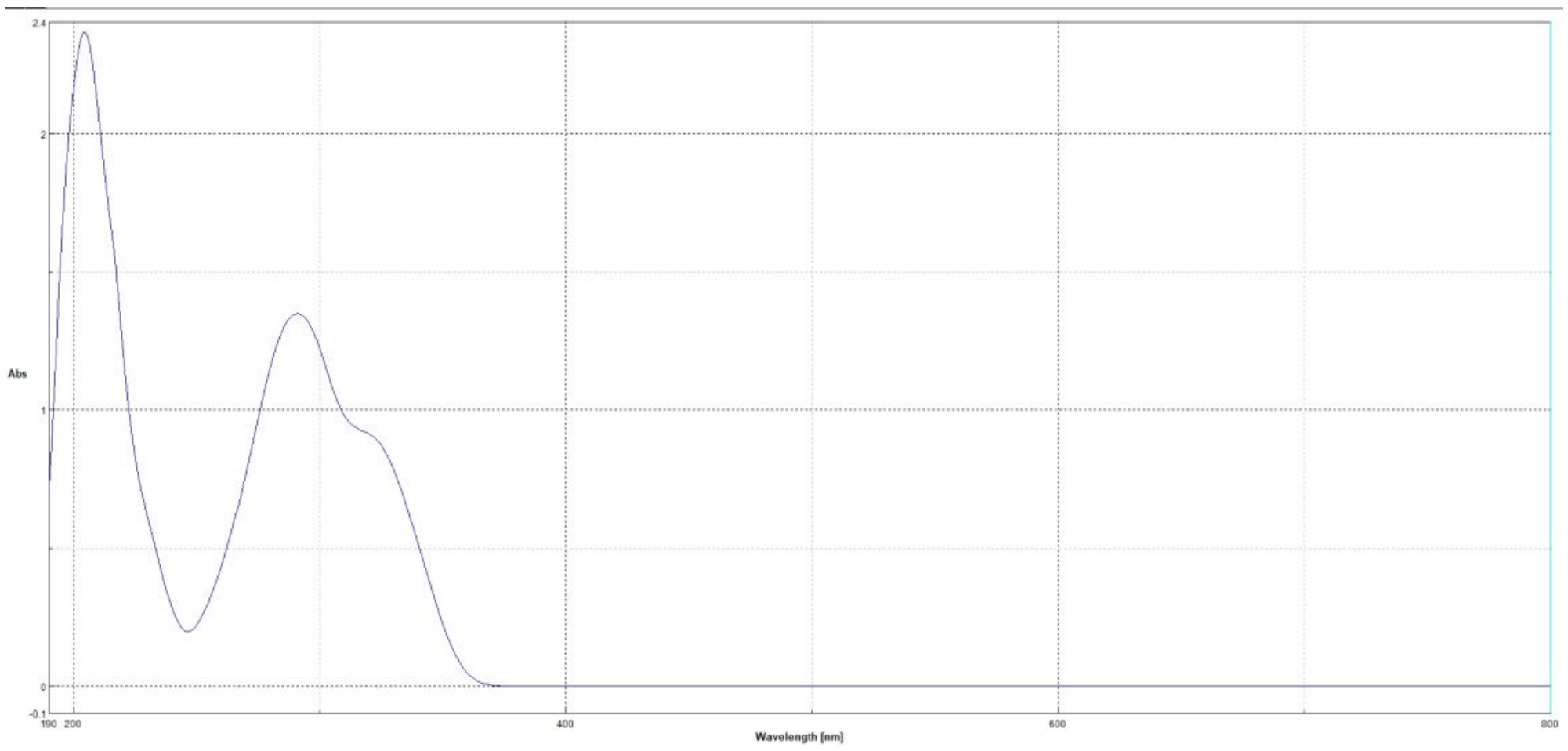

Figure S3. UV-Vis spectrum of a 10^{-4} M solution of 3-CCA at pH 7.4.

S5

Fluorescence spectra of the solutions separated from CRO, CHR and ERI at different times

Figures S4a, S4b and **S4c** report the fluorescence spectra of the 7-OH-3-CCA species produced by CRO, CHR and ERI fibers reacted with 3-CAA 10^{-4} M and H_2O_2 2×10^{-4} M at pH 7.4 as a function of time showing the emission signal at 446 nm ($I_{em\ 446\ nm}$) whose intensity is plotted in Figure 1 of the main text.

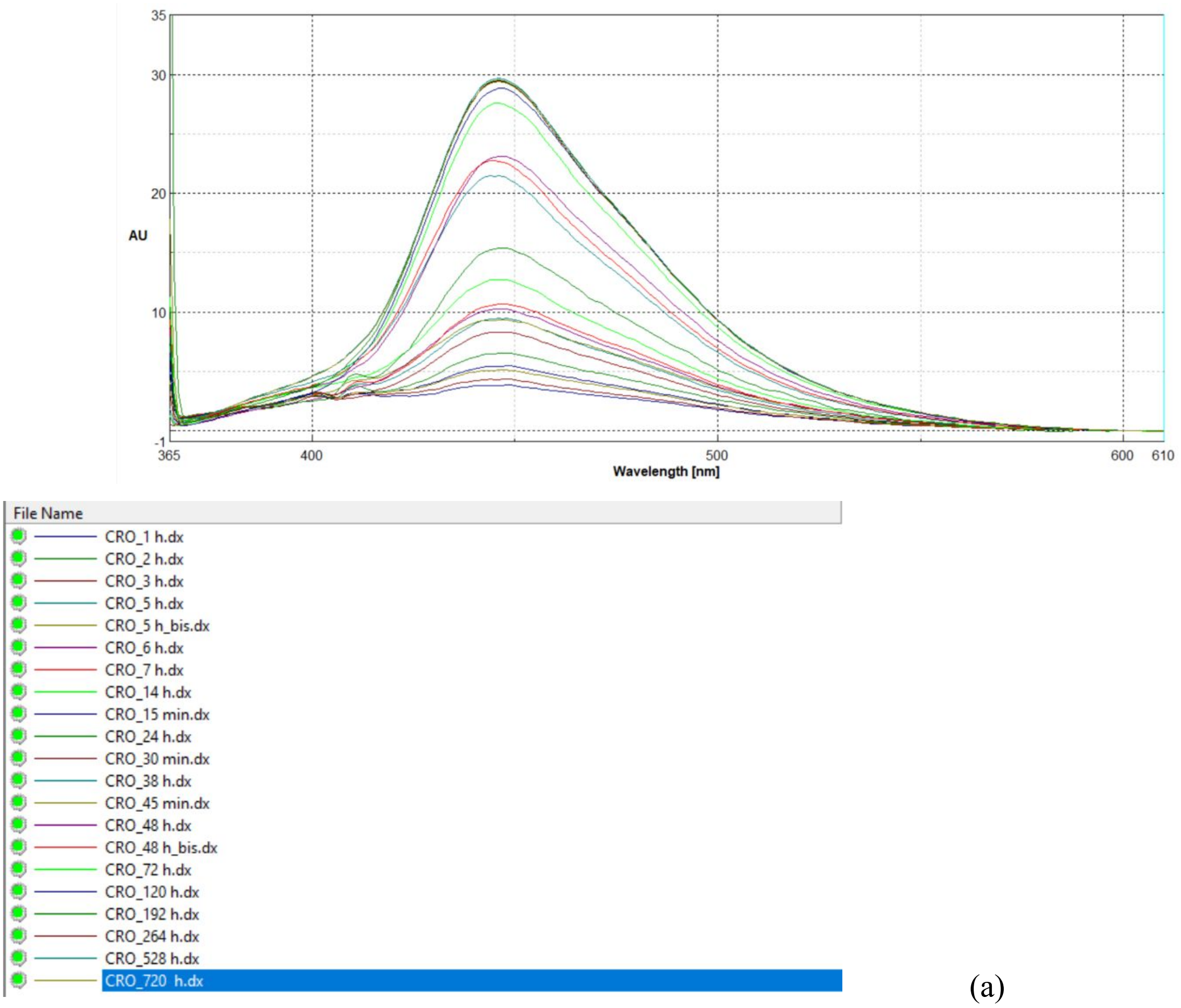

(a)

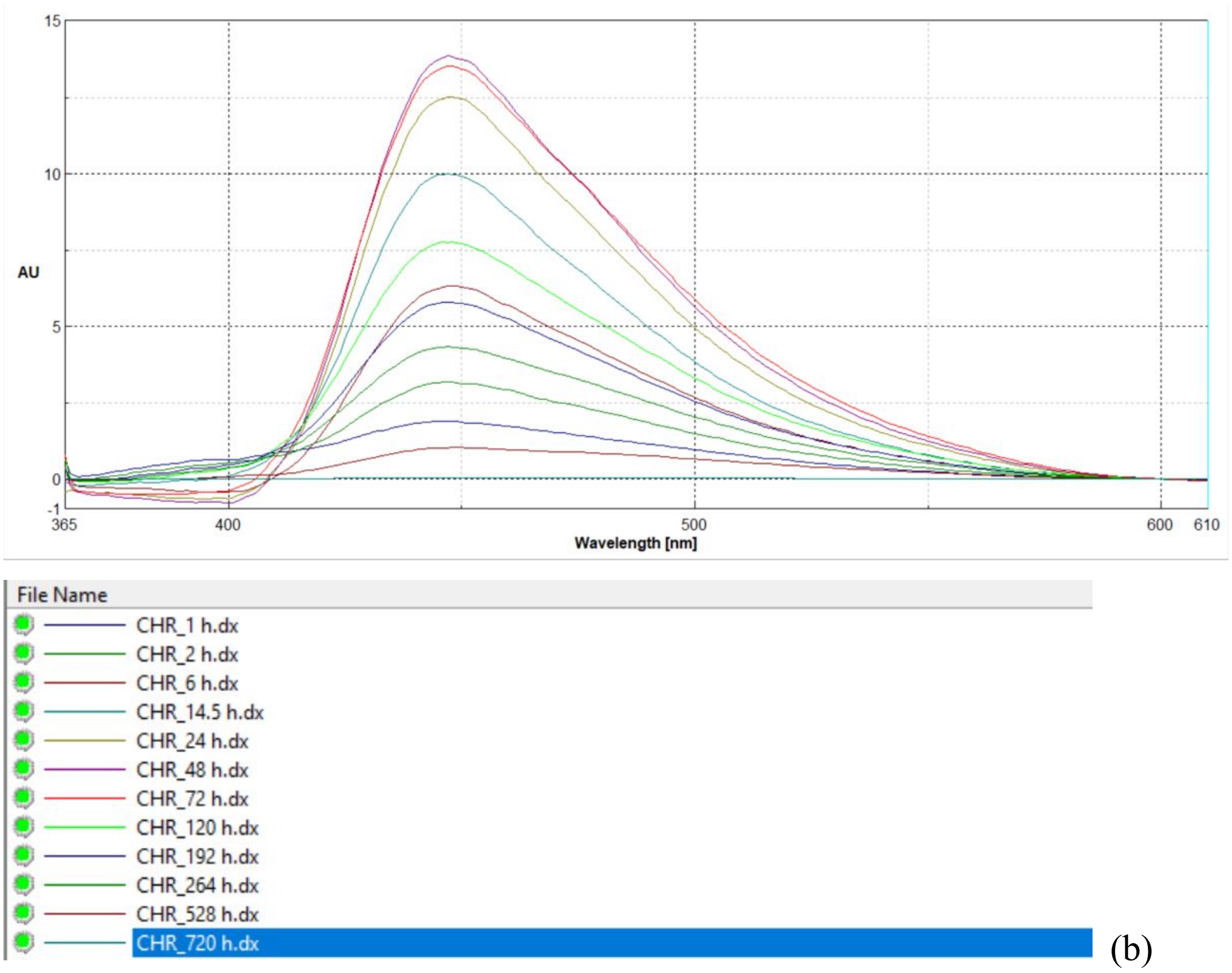

(b)

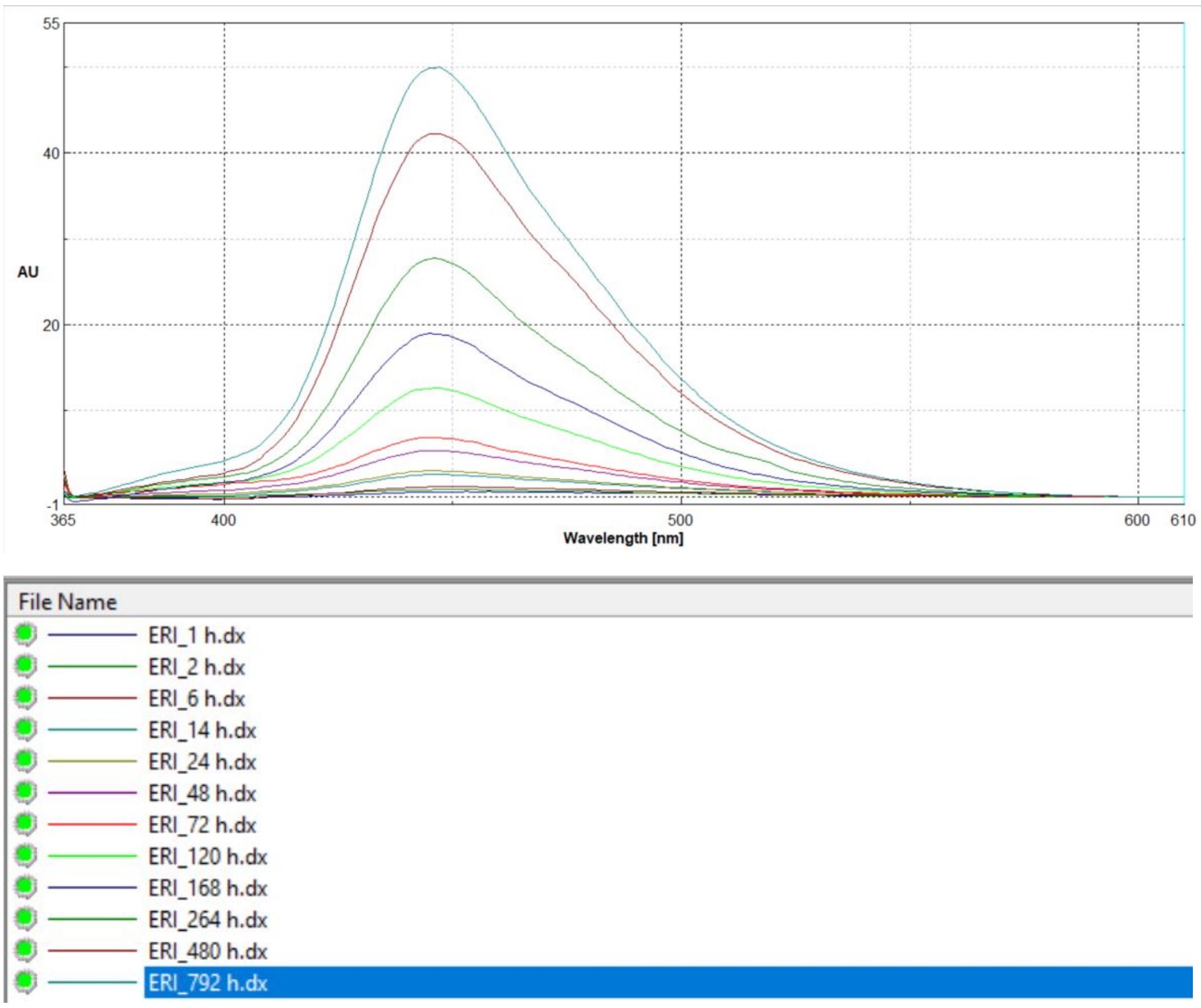

(c)

Figure S4. Fluorescence spectra of the solutions separated from CRO (a), CHR (b) and ERI (c) reacted with 10^{-4} M 3-CCA and 2 x 10^{-4} M H_2O_2 at pH 7.4 at different times (a).

S6

Time course of the intensity of the fluorescence emission of the 7-OH-3-CCA species produced by fibers without and with TEMPO scavenger.

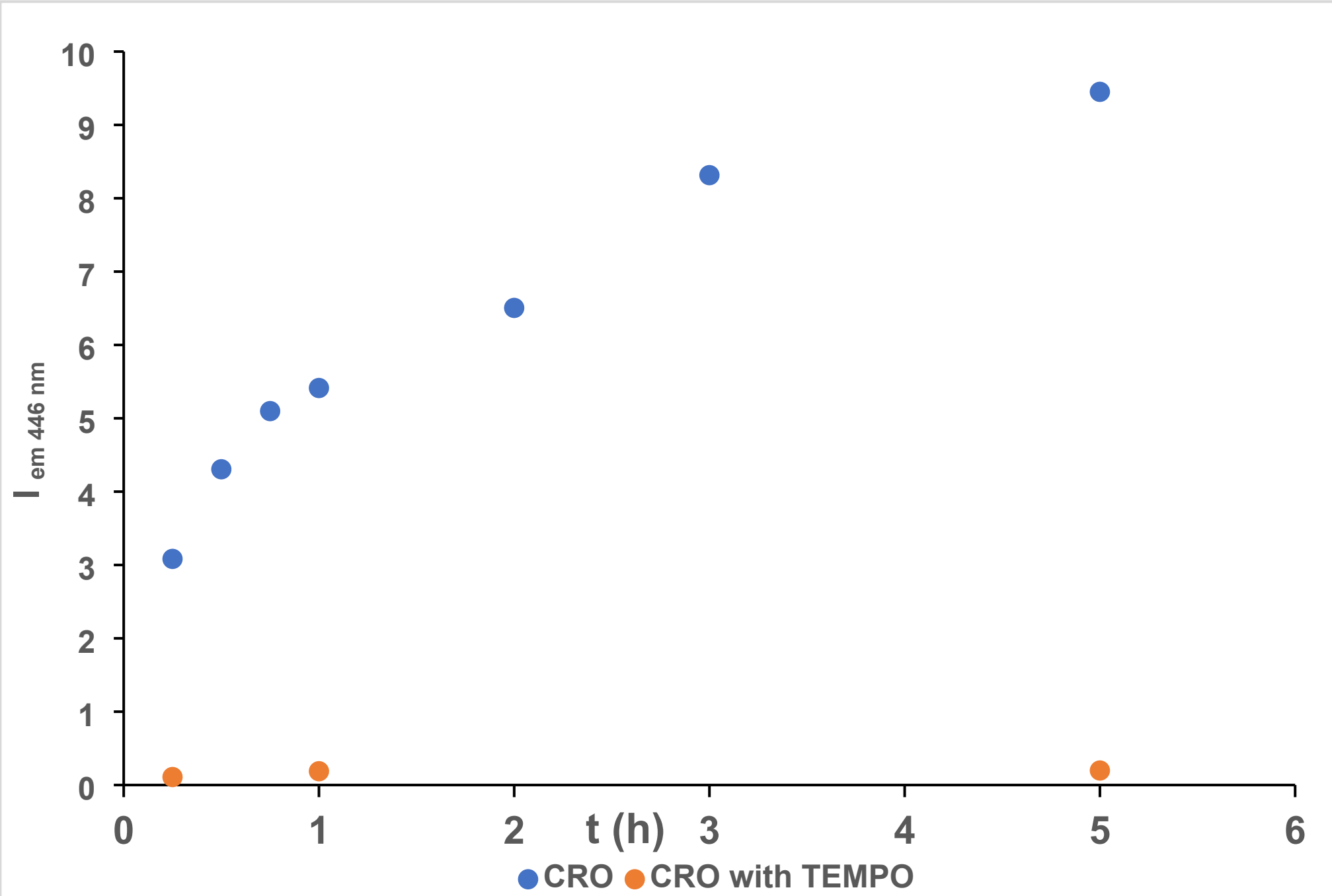

(a)

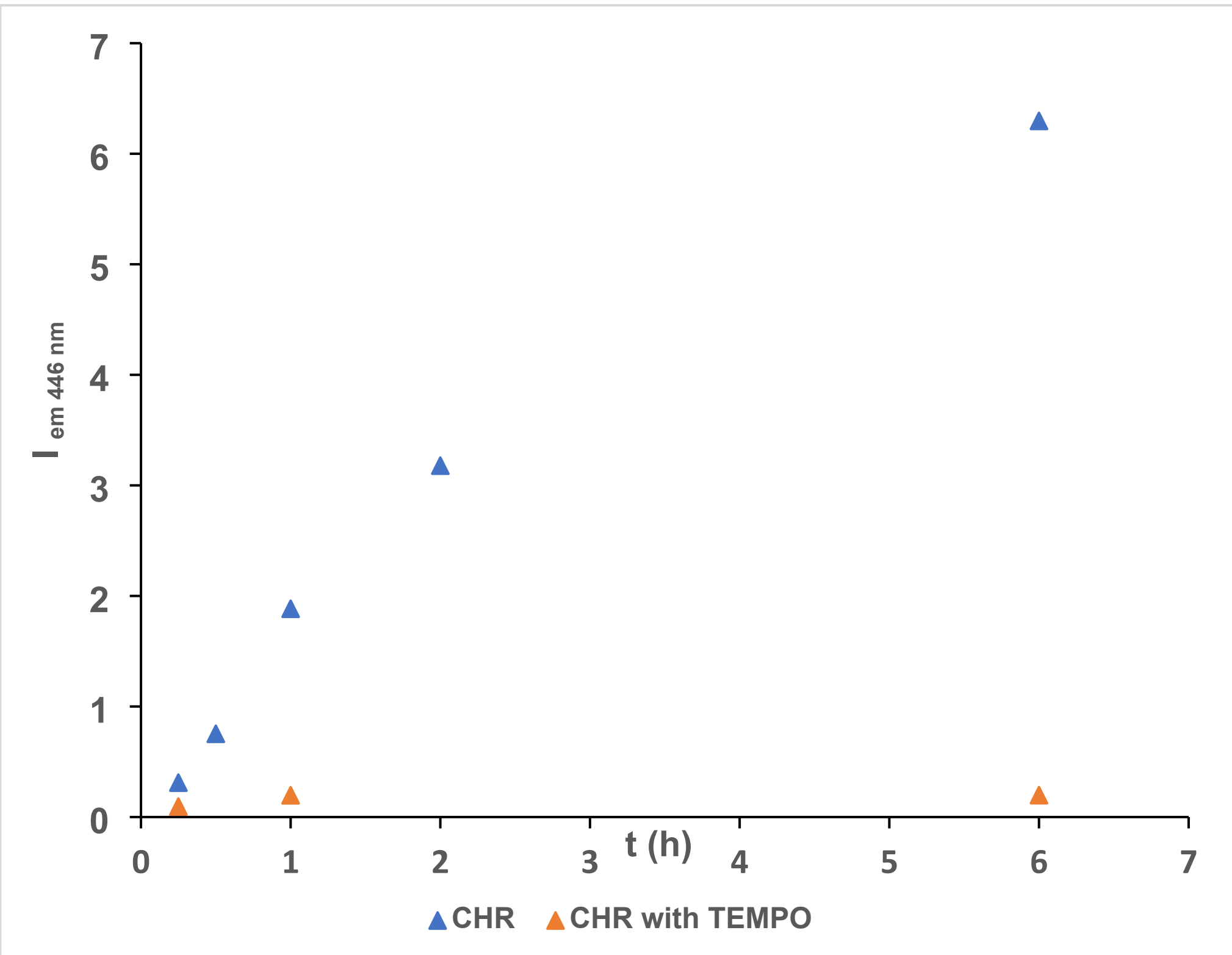

(b)

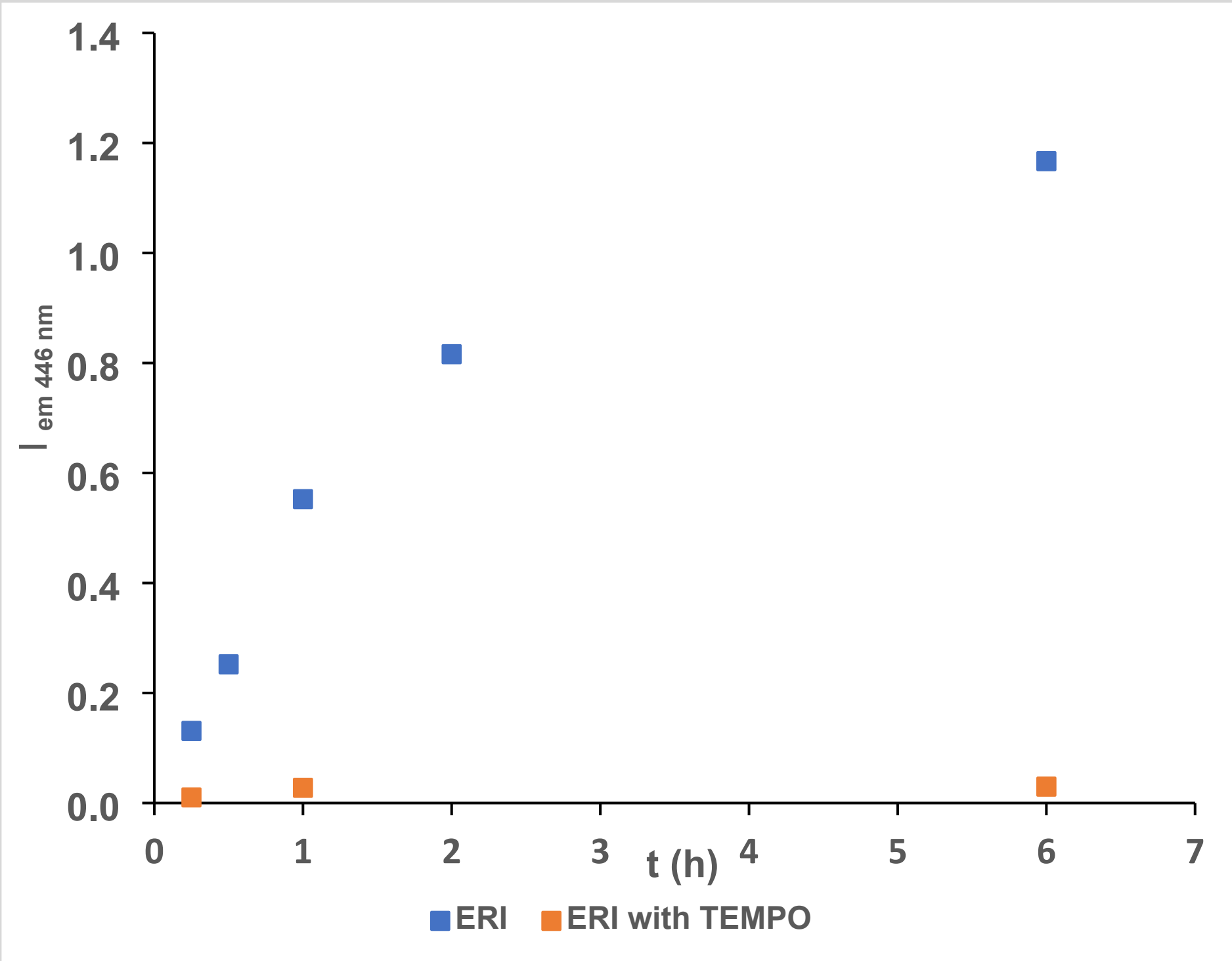

(c)

Figure S5. Time course of the intensity of the fluorescence emission signal at 446 nm ($I_{em\ 446\ nm}$) of the 7-OH-3-CCA species produced by CRO (a), CHR (b) and ERI (c) fibers reacted with 10^{-4} M 3-CCA and $2x10^{-4}$ M H_2O_2 at pH 7.4 without (blue) and with (orange) 10^{-3} M TEMPO scavenger.

S7

UV-Vis spectra of the solutions separated from CRO and ERI at different times

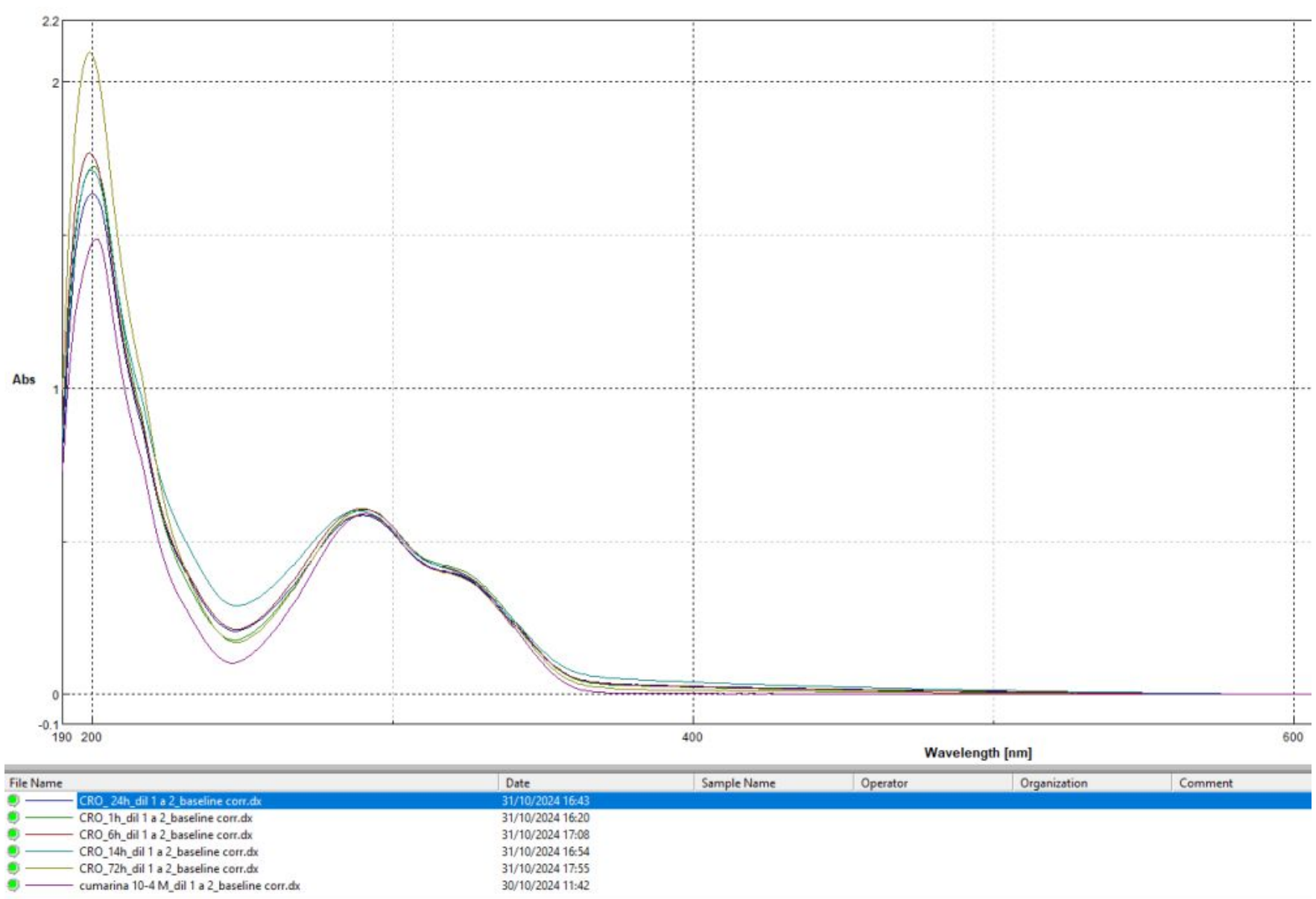

(a)

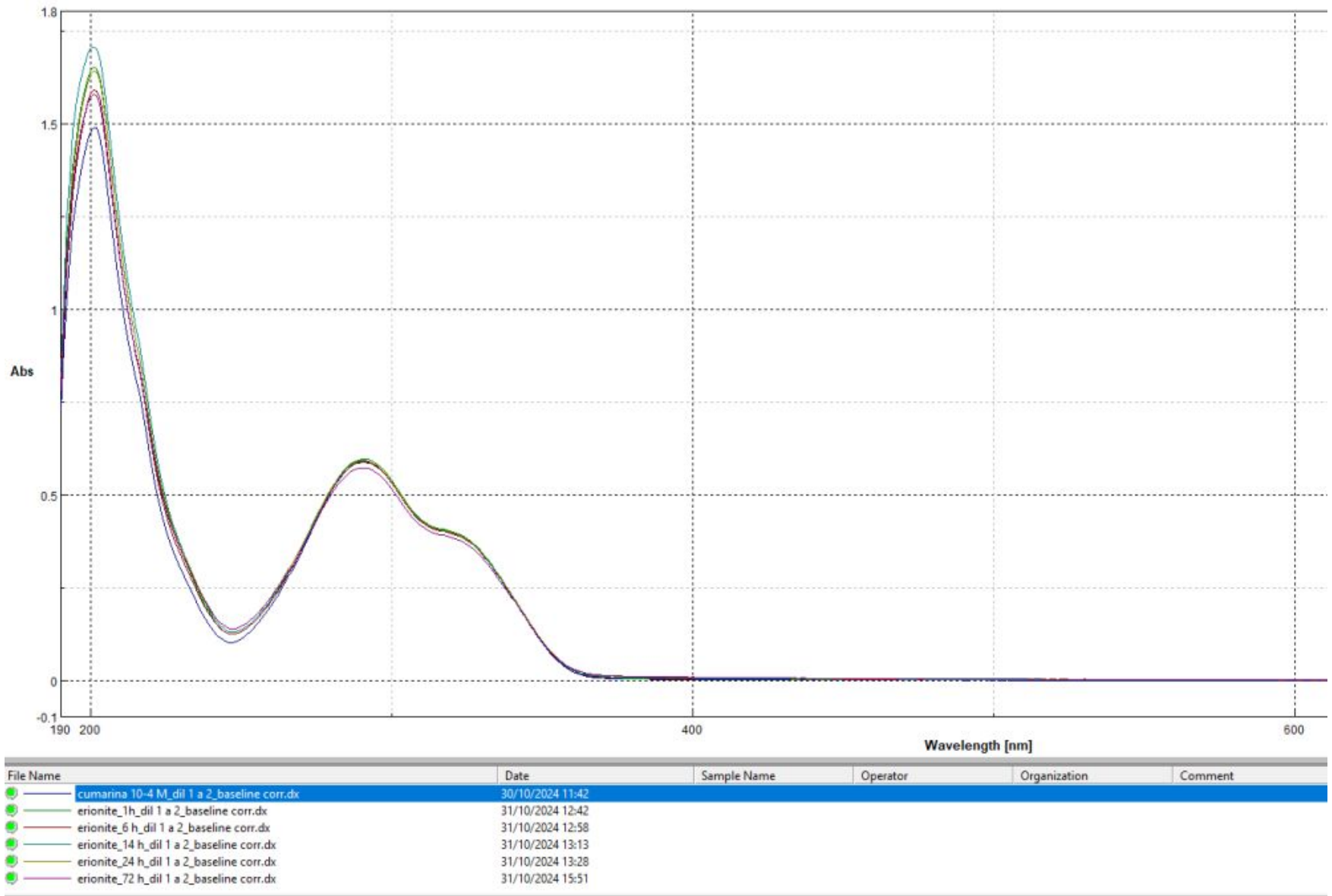

(b)

Figure S6. UV-Vis spectra of the starting 3-CCA 10^{-4} M solution diluted 1:2 (purple) and of the solutions separated from the suspensions made by CRO plus 10^{-4} M 3-CCA and 2×10-4 M H_2O_2 at 1 (green), 6 (brown), 14 (yellow), 24 (dark green), and 72 (rotten green) h, diluted 1:2 (a). UV-Vis spectra of the starting 3-CCA 10^{-4} M solution diluted 1:2 (blue) and of the solutions separated from the suspensions made by ERI plus 10^{-4} M 3-CCA and 2×10^{-4} M H_2O_2 at 1 (green), 6 (brown), 14 (dark green), 24 (rotten green), and 72 (purple) h diluted, 1:2 (b).

S8

3-CCA-H_2O_2-fluorescence method applied to leachate solutions to detect homogeneous Fenton contribution

Figure S7 reports the results of the 3-CCA-H_2O_2-fluorescence method applied directly on separated, fiber-free leachates after 1, 3, 14 days of contact with 10^{-4} M 3-CCA solution at pH 7.4 and T = 37°C.

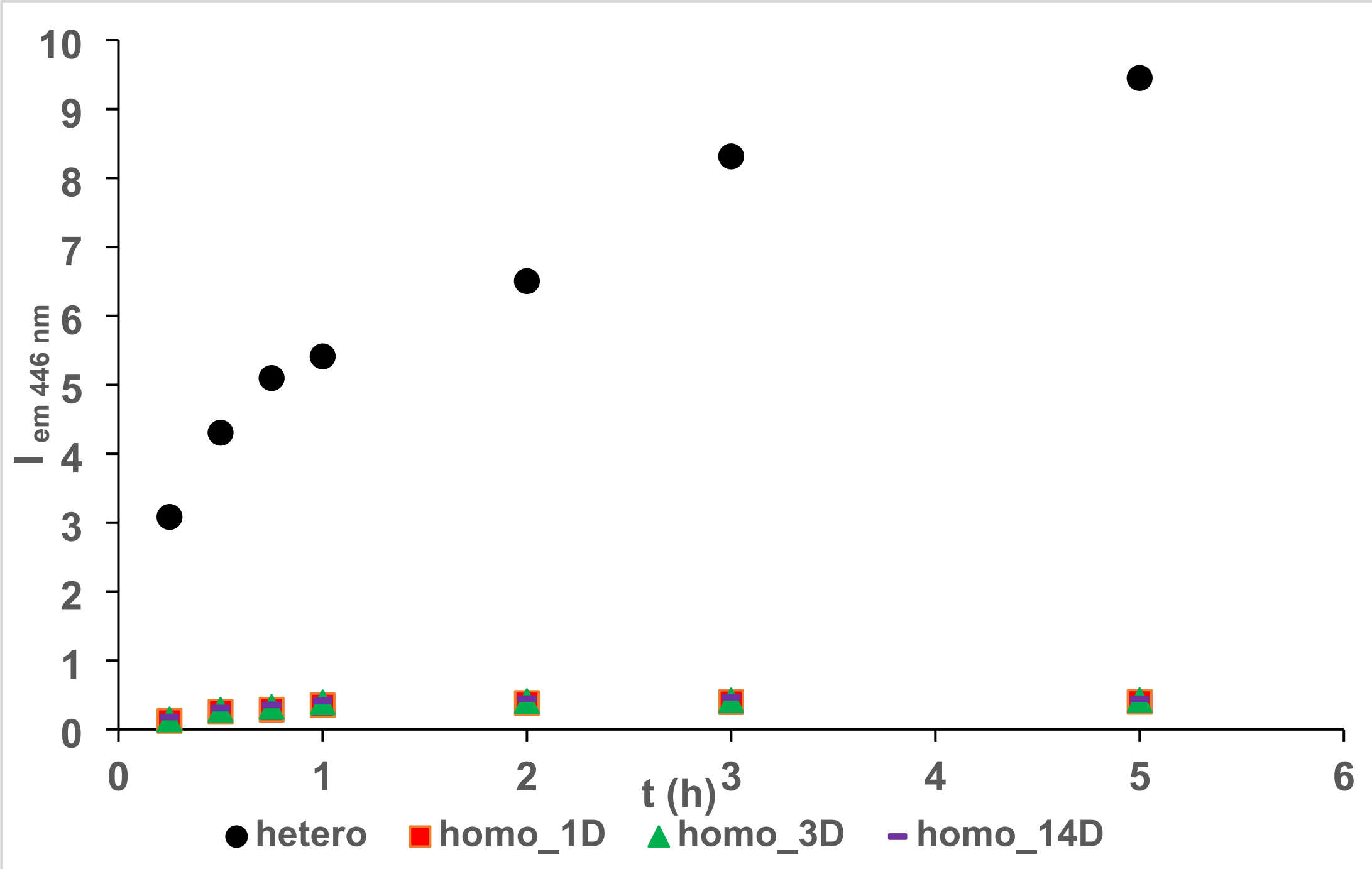
10
9
8
7
6
5
4
3
2
1
0
I em 446 nm
0
1
2
t (h)
3
4
5
6
hetero
homo_1D
homo_3D
homo_14D

(a)

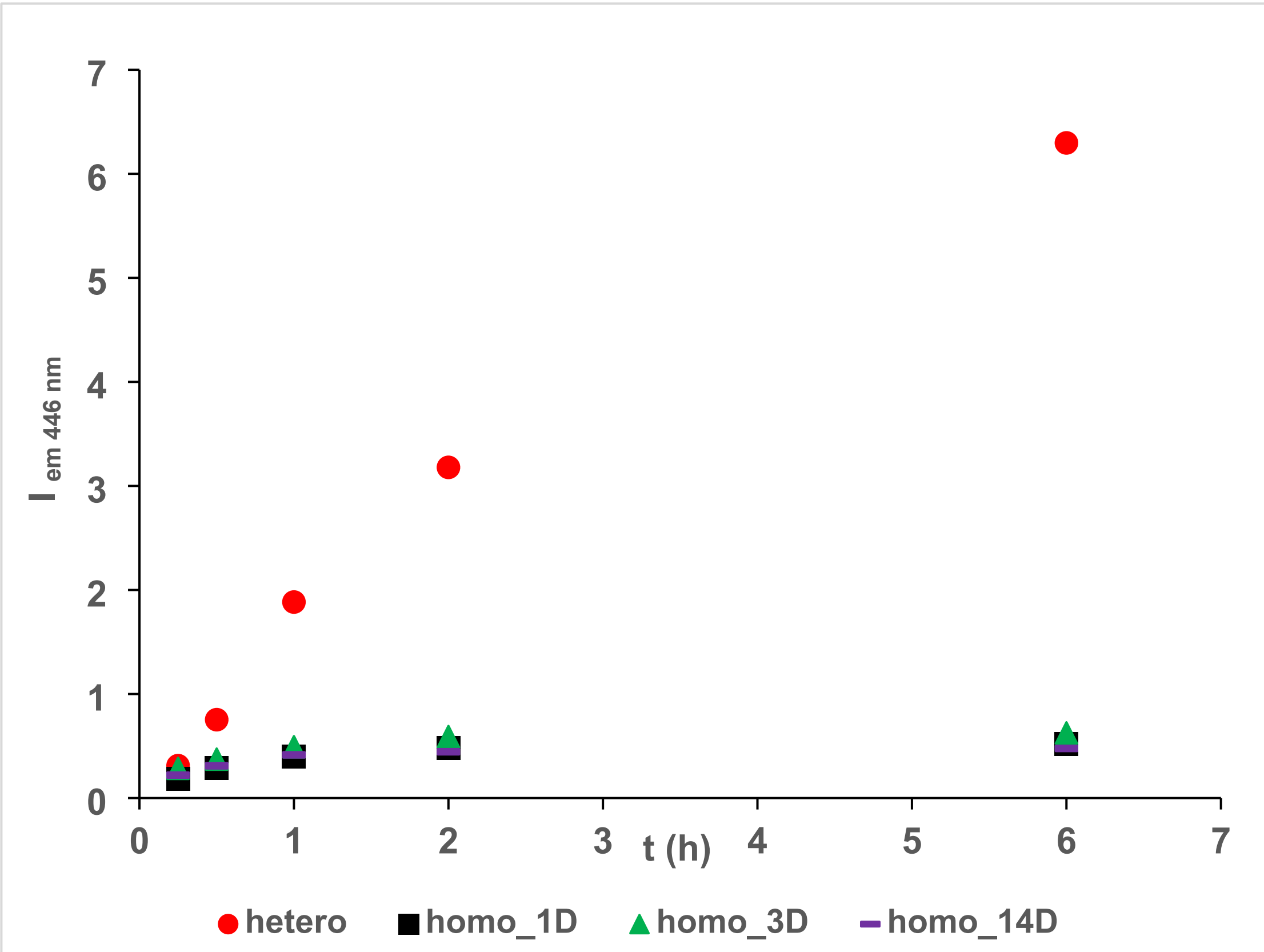
7
6
5
4
3
2
1
0
I em 446 nm
0
1
2
3
t (h)
4
5
6
7
hetero
homo_1D
homo_3D
homo_14D

(b)

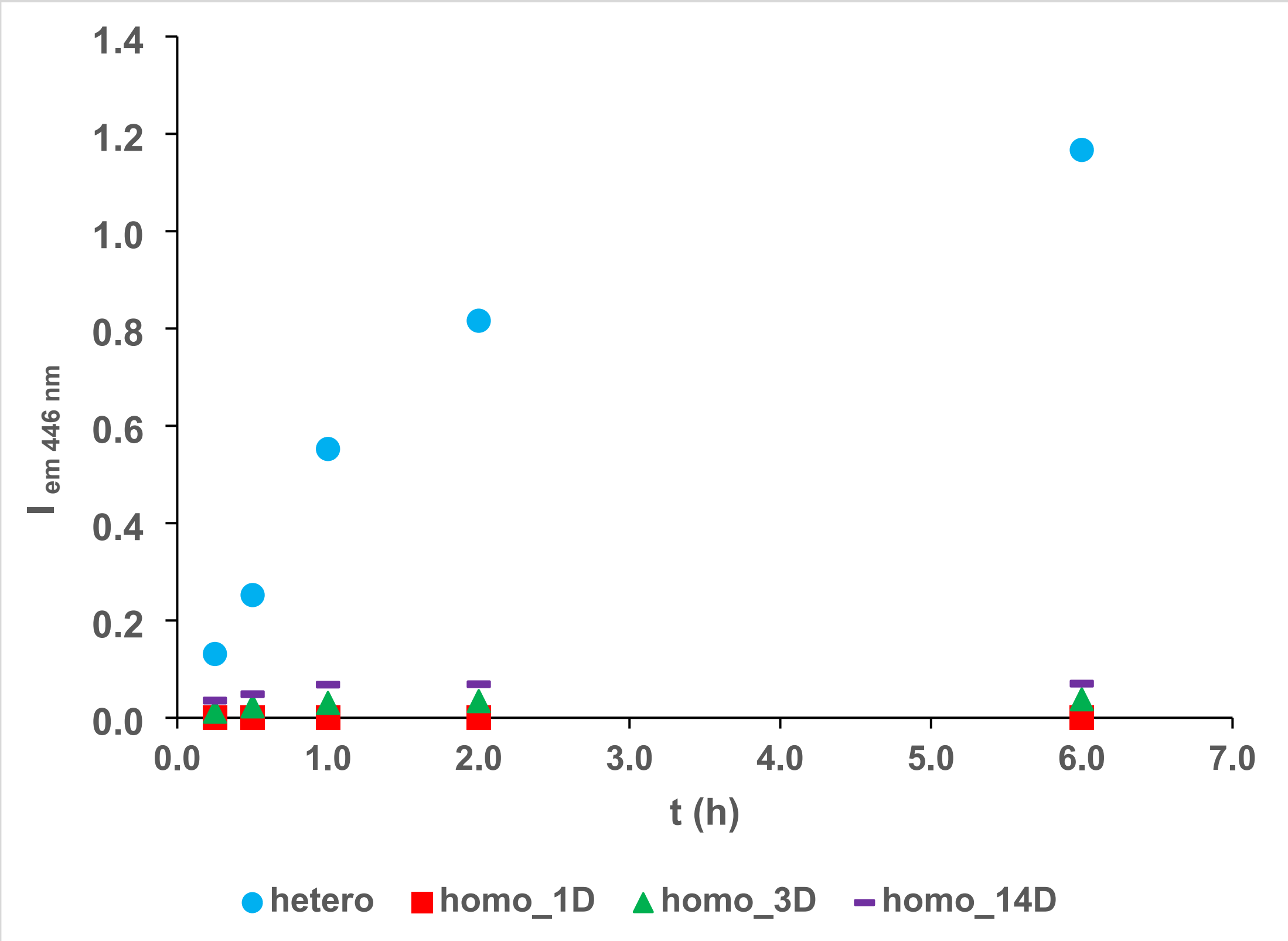

(c)

Figure S7. Time course of the intensity of the fluorescence emission signal at 446 nm ($I_{em\ 446\ nm}$) of the 7-OH-3-CCA species in the heterogeneous system (taken from Figure 1) compared with the same signal produced in leachate solutions from CRO (a), CHR (b) and ERI (c) fibers after 1, 3, 14 days of contact with 10^{-4} M 3-CCA solution at pH 7.4 and T = 37°C and tested with the 3-CCA-H_2O_2-fluorescence method (homogeneous system, homo_1D, homo_3D, homo_14D).

S9

X-ray powder diffraction (XRPD)

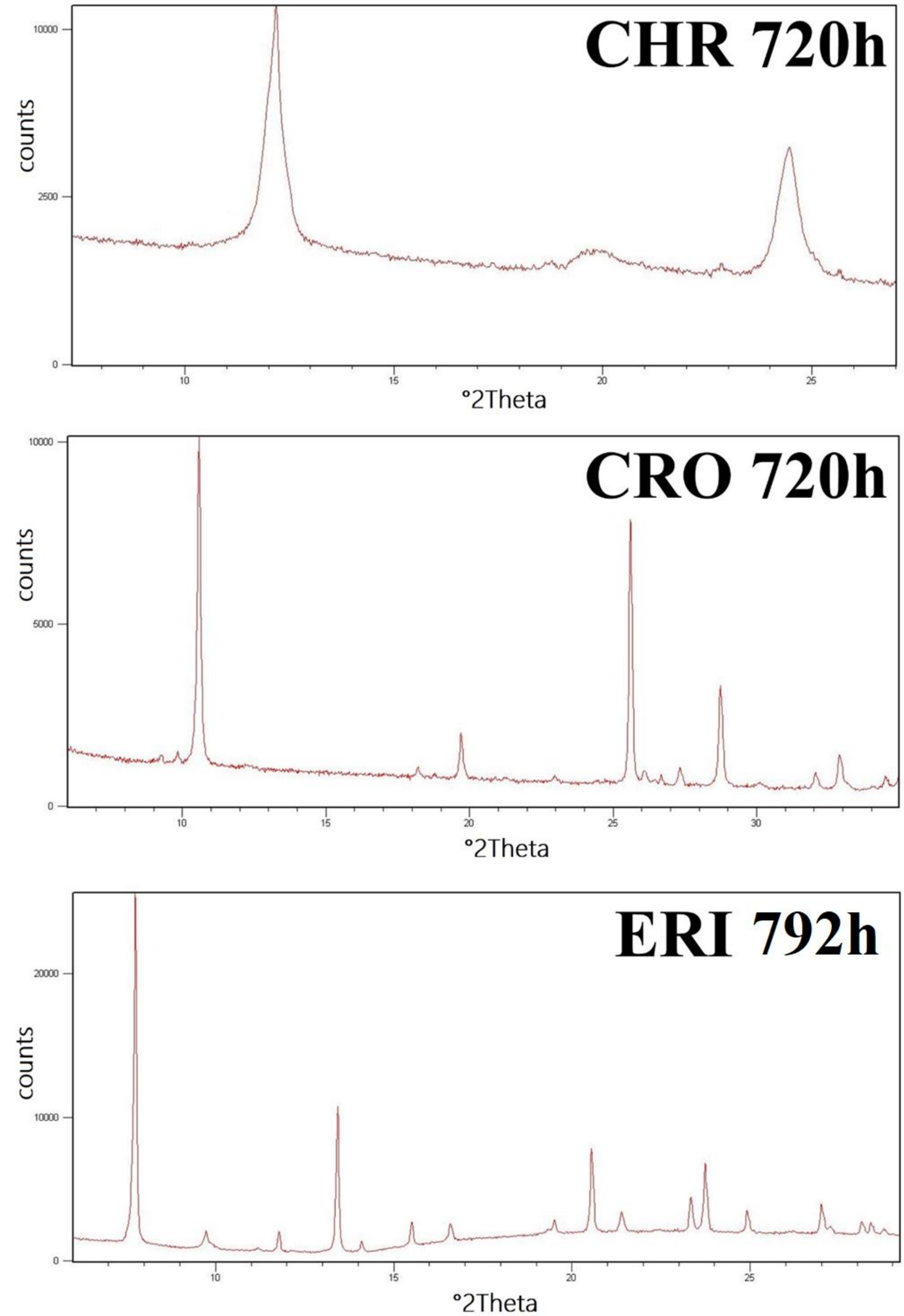

Figure S8. X-ray powder diffraction patterns of the solid fraction separated from the solution containing the reaction products of CHR, CRO and ERI at pH 7.4 after at 720h (CHR an CRO) and 792h (ERI) witnessing the stability of the three minerals.

References

(1) Smith, M.T.; Guyton, K.Z.; Gibbons, C.F.; Fritz, J.M.; Portier, C.J.; Rusyn, I.; De Marini, D.M.; Caldwell, J.C.; Kavlock, R.J.; Lambert, P.F.; Hecht, S.H.; Bucher, J.R.; Stewart, B.W.; Baan, R.A.; Cogliano, V.J.; Straif, K. Key characteristics of carcinogens as a basis for organizing data on mechanisms of carcinogenesis. *Env. Health Persp.* **2016**, *124(6)*, 713-721. Doi: 10.1289/ehp.1509912

(2) Gualtieri, A.F.; Cocchi, M.; Muniz-Miranda, F.; Pedone, A.; Castellini, E.; Strani, L. Iron Nuclearity in Mineral Fibers: Unravelling the Catalytic Activity for Predictive Modelling of Toxicity. *J. Haz. Mater.* **2024**, *469*, 134004. Doi.org/10.1016/j.jhazmat.2024.134004

(3) Di Giuseppe, D., Zoboli, A., Nodari, L., Pasquali, L., Sala, O., Ballirano, P., Malferrari, D., Raneri, S., Hanuskova, M., Gualtieri, A.F., 2021. Characterization and assessment of the potential toxicity/pathogenicity of Russian commercial chrysotile. *American Mineralogist*, 106, 1606-1621. Doi.org/10.2138/am-2021-7710

(4) Pollastri, S.; Gualtieri, A.F.; Lassinanntti Gualtieri, M.; Hanuskova, M.; Cavallo, A.; Gaudino, G. The zeta potential of mineral fibers. *J. Haz. Mater.* **2014**, *276*, 469-479. Doi.org/10.1016/j.jhazmat.2014.05.060

(5) Degen, T.; Sadki, M.; Bron, E.; Konig, U.; Nenert, G. The HighScore suite. *Powder Diffr.* **2014**, *29*. Doi.org/10.1017/S0885715614000840
